# Rapid-CNS^2^: rapid comprehensive adaptive nanopore-sequencing of CNS tumors, a proof-of-concept study

**DOI:** 10.1007/s00401-022-02415-6

**Published:** 2022-03-31

**Authors:** Areeba Patel, Helin Dogan, Alexander Payne, Elena Krause, Philipp Sievers, Natalie Schoebe, Daniel Schrimpf, Christina Blume, Damian Stichel, Nadine Holmes, Philipp Euskirchen, Jürgen Hench, Stephan Frank, Violaine Rosenstiel-Goidts, Miriam Ratliff, Nima Etminan, Andreas Unterberg, Christoph Dieterich, Christel Herold-Mende, Stefan M. Pfister, Wolfgang Wick, Matthew Loose, Andreas von Deimling, Martin Sill, David T. W. Jones, Matthias Schlesner, Felix Sahm

**Affiliations:** 1 Department of Neuropathology, University Hospital Heidelberg, Heidelberg, Germany; 2 Clinical Cooperation Unit Neuropathology, German Cancer Consortium (DKTK), German Cancer Research Center (DKFZ), Im Neuenheimer Feld 224, 69120 Heidelberg, Germany; 3 DeepSeq, School of Life Sciences, University of Nottingham, Nottingham, UK; 4 Department of Neurology, Charité-Universitätsmedizin Berlin, Berlin, Germany; 5 Division of Neuropathology, Institute of Pathology, University Hospital Basel, Basel, Switzerland; 6 Brain Tumor Translational Targets, German Cancer Research Center (DKFZ), Heidelberg, Germany; 7 Department of Neurosurgery, University Hospital Mannheim, Mannheim, Germany; 8 Department of Neurosurgery, University Hospital Heidelberg, Heidelberg, Germany; 9 Department of Cardiology, Angiology, and Pneumology, University Hospital Heidelberg, University of Heidelberg, Heidelberg, Germany; 10 Hopp Children’s Cancer Center (KiTZ), Heidelberg, Germany; 11 Division of Pediatric Neurooncology, German Cancer Research Center (DKFZ), German Cancer Consortium (DKTK), Heidelberg, Germany; 12 Department of Pediatric Hematology and Oncology, Heidelberg University Hospital, Heidelberg, Germany; 13 Pediatric Glioma Research Group, German Cancer Research Center (DKFZ), Heidelberg, Germany; 14 Clinical Cooperation Unit Neurooncology, German Consortium for Translational Cancer Research (DKTK), German Cancer Research Center (DKFZ), Heidelberg, Germany; 15 Department of Neurology and Neurooncology Program, National Center for Tumor Diseases, Heidelberg University Hospital, Heidelberg, Germany; 16 Biomedical Informatics, Data Mining and Data Analytics, Augsburg University, Augsburg, Germany

Molecular markers are now unequivocally a requirement for integrative brain tumor diagnostics. The 2021 WHO classification of CNS tumors substantially increases the set of genes required in routine evaluation, and significantly increases the relevance of DNA methylation analysis [12]. The advantages of custom neuropathology NGS panels can only be efficiently exploited when case numbers are sufficient for batchwise processing. Labs with lower specimen submission numbers hence may have to pool samples over multiple weeks. Here we introduce Rapid-CNS^2^—a custom neurooncology molecular diagnostic workflow using Nanopore “third generation” sequencing for parallel copy-number profiling, mutational and methylation analysis that is highly flexible in target selection, runs efficiently on single samples, and can be initiated immediately upon receipt of frozen sections.

Nanopore sequencing has an advantage over current NGS in read lengths, shorter and easier library preparation, ability to call base modifications, real time analysis, and portability of sequencing devices—all at relatively low cost [2]. However, smaller devices like the MinION and GridION yield low-coverage data when run genome-wide [6]. While nanopore-based approaches to methylation classification, particularly nanoDx, have demonstrated reliable methylation classification with as low as 1000 CpGs, it does not allow for detection of single-nucleotide-variants (SNVs) or *MGMT* promoter methylation status assessment [4, 7]. Intriguingly, Nanopore provides a “ReadUntil” adaptive sampling toolkit that can reject reads in real-time during sequencing [8]. The tool “ReadFish” harnesses this functionality to enable targeted adaptive sequencing with no additional steps in library preparation, which has been complemented by a built-in adaptive sampling in recent device versions [10]. This considerably increases coverage over “target” regions by real-time enrichment during sequencing.

Rapid-CNS^2^ leverages adaptive nanopore sequencing through ReadFish and the built-in adaptive sampling and is run here as a proof-of-concept on a portable MinION or GridION device, respectively. We formulated target regions covering our brain tumor NGS panel and CpG sites for methylation classification [4, 13]. Sequenced data was analyzed using an end-to-end bioinformatics pipeline that reports clinically relevant SNVs, copy number alterations, target gene methylation and methylation classification for CNS tumors. Methylation classification was based on nanoDx’s ad-hoc approach but infers CpG importance from the Heidelberg methylation classifier and was trained on the published reference set [3]. *MGMT* promoter methylation status was estimated by a proposed logistic regression-based prediction model (Supplementary Methods, online resource).

We first sequenced 45 cryoconserved glioma samples for 72 h each to investigate the overall feasibility for detecting relevant alterations (Fig. 1). Targets (Rapid_CNS_A) included the regions of the aforementioned panel and 10,000 CpG sites inferred from the Heidelberg methylation classifier (155 Mb). Molecular alterations were compared to NGS panel sequencing and EPIC array analyses (referred to as “conventional data/analysis”) from corresponding FFPE tissue samples [1, 3, 13]. Incubation time and other parameters were optimized to improve quality, amount of data generated and on-target rate of the libraries (Supplementary Methods, online resource). Samples were selected to cover a variety of clinically relevant pathognomonic alterations (including but not limited to *IDH1*, 1p/19q codeletion, chr7 gain/chr10 loss, *TERT* promoter, *EGFR* amplification, *CDKN2A/B* deletion, *MGMT* status) and relevant methylation classes. Second, a smaller panel was developed to compare the effect of target sizes. Rapid_CNS_B comprised regions for mutational analysis only (15 Mb) and was run on 8 glioma samples (Supplementary Fig. 1, online resource). Third, we assessed the reusability of flow cells and the data quality of shorter sequencing times by splitting 5 of the Rapid_CNS_B samples into 24 h and 48 h run intervals.

The glioma samples run with the Rapid_CNS_A panel as target region were each loaded onto FLO-MIN106 R9.4.1 flow cells and sequencing was controlled in real-time by ReadFish (using a GPU powered consumer notebook). Targeted regions showed an average coverage of 15X. (Supplementary Fig. 1, online resource). *IDH1* and *TERTp* mutation status were correctly identified in 44/45 and 43/45 glioma samples, respectively. Upon re-evaluation of histology and raw data, discrepancy in mutations could be attributed to low tumor cell content in the respective cryopreserved sections in two cases, and variant filtering in another (Supplementary Results, online resource). Copy number alterations displayed complete concordance with EPIC array results and markedly better resolution as compared to NGS panel sequencing (Supplementary Fig. 2, online resource). *MGMT* promoter status was concordant with both pyrosequencing and EPIC array in all cases (Supplementary Fig. 3, online resource). Methylation families (relevant for WHO-conform diagnosis) were identified in line with the Heidelberg classifier predictions for all samples, with even the methylation subclass correctly identified for 37/45 glioma samples (Fig. 1d).

**Fig. 1:**
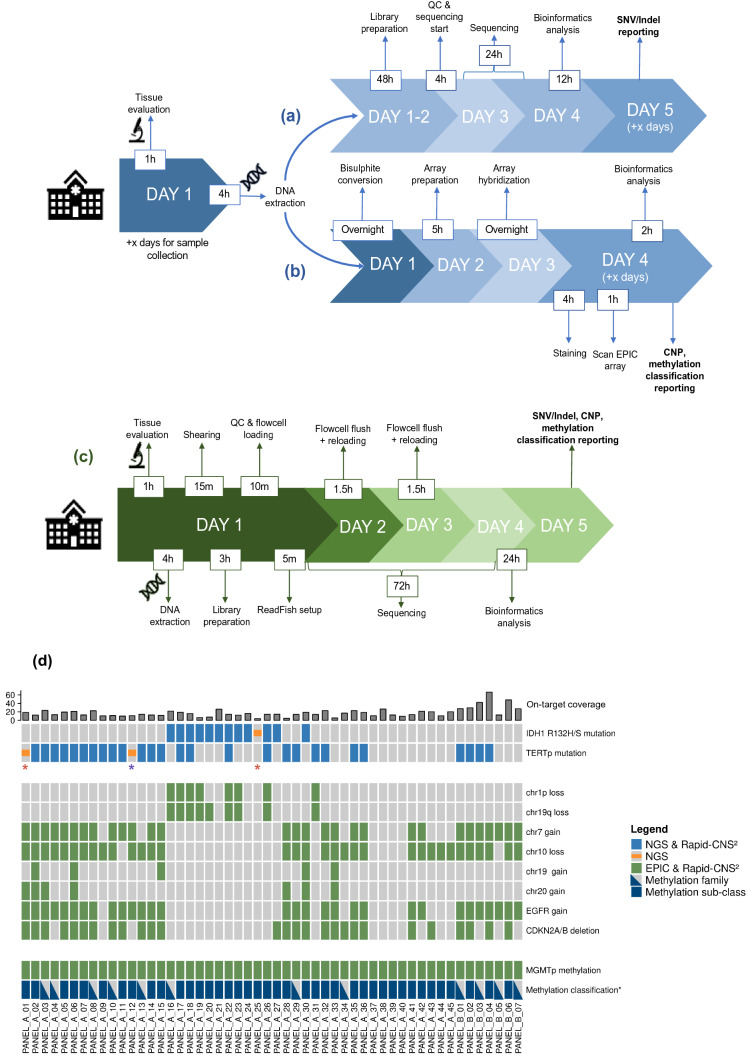
Rapid-CNS^2^ timeline and concordance with standard methods. Timeline for **a** NGS panel sequencing and analysis pipeline, and **b** EPIC array analysis pipeline for neuropathology diagnostics (x denotes number of days required to pool sufficient samples). **c** Timeline for Rapid-CNS^2^ sequencing and analysis for a single sample on a MinION or up to five samples on a GridION. **d** Concordance of clinically relevant alterations and classification for diffuse glioma samples with matching NGS panel sequencing and EPIC array analysis. Colored blocks indicate presence of alteration, concordance for detected alterations is denoted in the legend. Triangular denotations for methylation class indicate samples where methylation families were concordant and blocks indicate concordance for sub-classes as well. ‘PANEL_A’ indicates samples sequenced using Rapid_CNS_A, and ‘PANEL_B’ indicates samples sequenced using Rapid_CNS_B (sample by Rapid-CNS^2^ and 850 k identified as AT/RT excluded, depicted in Supplementary Fig. 4, online resource). Top barplot indicates mean on-target coverage. Red asterisk—different tissue region for Rapid-CNS^2^ vs conventional analyses, purple asterisk—alteration filtered by PEPPER-Margin-DeepVariant (see main text for details)

Reducing the target size with the Rapid_CNS_B panel doubled the mean on-target coverage to 39X with 3.4X average coverage over off-target regions (Supplementary Fig. 1, online resource). *IDH1*, *TERTp*, and *MGMT* status matched Rapid-CNS^2^ in all eight cases. Methylation family was correctly predicted in all cases, with accurate subclass predictions in 4/8 cases. Despite the reduction in targeted regions, copy number profile resolution was maintained and was concordant with EPIC array for all cases. One sample identified as a glioma by histology was correctly classified as an atypical teratoid/rhabdoid tumor by Rapid-CNS^2^ (confirmed by EPIC array analysis, Supplementary Fig. 4, online resource).

The histopathological suspicion for diffuse glioma in six additional prospective cases was confirmed by Rapid-CNS^2^ (Rapid_CNS_A: 2 samples, Rapid_CNS_B: 4 samples) in a real-time clinical setting with respective reports issued (Supplementary Fig. 5, online resource). *MGMT* promoter status for these samples was in line with pyrosequencing.

Shortening sequencing time to 24 h was sufficient for a comprehensive diagnosis. The flowce ll could also be reliably reused for another sample for the next 48 h (Supplementary Fig. 6, online resource). Detection of clinically relevant fusions and complex genomic rearrangements using targeted Nanopore DNA sequencing still requires considerable manual intervention [5, 9]. As such, automated detection of such alterations is a developing field, and hence beyond the current scope of this study. In its current state, our attempts on FFPE derived DNA showed that Rapid-CNS^2^ is restricted to use with cryopreserved or fresh tumor samples (Supplementary Fig. 7, online resource). Considering the potential for further advances in Nanopore technology, FFPE sample processing may become feasible in the future.

Targeted regions for Rapid-CNS^2^ can be easily altered by editing a BED file, in principle allowing even much lower sequencing times than in this study. The recently introduced barcode-aware adaptive sampling by ReadFish proposes an effective way to multiplex samples and further reduce costs [11]. With no additional library preparation steps required, it is possible to modify targeted regions for each individual sample as required. MinION is a portable, handheld device which makes it a rational option for smaller neuropathology labs or in lower-infrastructure locations. Collectively, the Rapid-CNS^2^ approach can be set-up at low capital expense, is cost-efficient (Supplementary Results, online resource) even in a low throughput setting, and provides a swift and highly flexible alternative to conventional methods for SNV/InDel analysis, *MGMT* promoter status, methylation classification and detection of copy number alterations.

## Supplementary Information

Below is the link to the electronic supplementary material.

Supplement ary file1 (PDF 16131 KB)
