# Supplementary material for: Rapid-CNS2: rapid comprehensive adaptive nanopore-sequencing of CNS tumors, a proof-of-concept study
Source: Acta Neuropathol. 2022 Mar 31;143(5):609–12. doi: 10.1007/s00401-022-02415-6 (PMC9038836; doi:10.1007/s00401-022-02415-6)
Supplement: Supplementary file 1 — Supplement ary file1 (PDF 16131 KB) [file 401_2022_2415_MOESM1_ESM.pdf]

Rapid-CNS^2: Rapid comprehensive adaptive nanopore-sequencing of CNS tumors, a proof-of-concept study

Areeba Patel[1,2]*, Helin Dogan[1,2]*, Alexander Payne[3], Elena Krause[1], Philipp Sievers[1,2], Natalie Schoebe[1,2], Daniel Schrimpf[1,2], Christina Blume[1,2], Damian Stichel[1,2], Nadine Holmes[3], Philipp Euskirchen[4], Jürgen Hench[5], Stephan Frank[5], Violaine Rosenstiel-Goidts[6], Miriam Ratliff[7], Nima Etminan[7], Andreas Unterberg[8], Christoph Dieterich[9], Christel Herold-Mende[8], Stefan M Pfister[10,11,12], Wolfgang Wick[14], Matthew Loose[3], Andreas von Deimling[1,2], Martin Sill[10,11]*, David TW Jones[10,13]*, Matthias Schlesner[15*], Felix Sahm[1,2,10]*

[1]Dept. of Neuropathology, University Hospital Heidelberg, Heidelberg, Germany.
[2]Clinical Cooperation Unit Neuropathology, German Cancer Consortium (DKTK), German Cancer Research Center, Heidelberg, Germany.
[3]DeepSeq, School of Life Sciences, University of Nottingham, Nottingham, United Kingdom,
[4] Department of Neurology, Charité-Universitätsmedizin Berlin, Berlin, Germany.
[5] Division of Neuropathology, Institute of Pathology, University Hospital Basel, Basel, Switzerland.
[6]Brain Tumor Translational Targets, German Cancer Research Center (DKFZ), Heidelberg, Germany.
[7]Dept. of Neurosurgery, University Hospital Mannheim, Mannheim, Germany.
[8]Dept. of Neurosurgery, University Hospital Heidelberg, Heidelberg, Germany.
[9]Department of Cardiology, Angiology, and Pneumology, University Hospital Heidelberg, University of Heidelberg, Heidelberg, Germany.
[10]Hopp Children's Cancer Center (KiTZ), Heidelberg, Germany.
[11]Division of Pediatric Neurooncology, German Cancer Research Center (DKFZ) and German Cancer Consortium (DKTK), Heidelberg, Germany
[12]Department of Pediatric Hematology and Oncology, Heidelberg University Hospital, Heidelberg, Germany
[13]Pediatric Glioma Research Group, German Cancer Research Center (DKFZ), Heidelberg, Germany
[14]Clinical Cooperation Unit Neurooncology, German Consortium for Translational Cancer Research (DKTK), German Cancer Research Center (DKFZ), Heidelberg, Germany and Department of Neurology and Neurooncology Program, National Center for Tumor Diseases, Heidelberg University Hospital, Heidelberg, Germany
[15]Biomedical Informatics, Data Mining and Data Analytics, Augsburg University, Augsburg, Germany

Corresponding author
Felix Sahm, MD, PhD
Department of Neuropathology
University Hospital Heidelberg
and
Clinical Cooperation Unit Neuropathology (B300)
German Cancer Research Center (DKFZ)
Im Neuenheimer Feld 224
69120 Heidelberg, Germany
Fon: +49-6221 56-37886
felix.sahm@med.uni-heidelberg.de

Supplementary Methods:

Samples

We sequenced 47 cryopreserved samples (45 archival, 2 prospective) targeting Rapid_CNS_A (details on target below), and 12 samples (8 archival, 4 prospective) targeting Rapid_CNS_B from the Department of Neuropathology, University Hospital Heidelberg (Suppl. Fig. 1d). Two additional FFPE samples, PANEL_A_48 (run for 72h) and PANEL_A_49 (stopped running at 24h due to poor sequencing) were also sequenced. (See Suppl. Table 1 for detailed overview of samples).

Library preparation:

Sections of 40x10 µm were prepared from cryoconserved tumor tissues with established molecular markers (IRB approval 2018-614N-MA, 005/2003) with tumor cell content (based on a H&E stain) > 60%. DNA was then extracted using the Promega Maxwell RSC Blood DNA Kit (catalogue # AS1400, Promega) on a Maxwell RSC 48 instrument (AS8500, Promega) per manufacturer's instructions. DNA concentrations were measured on a microplate reader (FLUOStar Omega, BMG Labtech) using the Invitrogen Qubit DNA BR Assay Kit (Q32851, Thermo Fisher Scientific). Next, the DNA was sheared to approximately 9 to 11 kb in a total volume of 50 µl using g-TUBEs (Covaris) at 7200 rpm for 120 sec. The fragment length was assessed on an Agilent 2100 Bioanalyzer (catalogue # G2939A, Agilent Technologies) with the Agilent DNA 12000 Kit (catalogue # 5067-1508, Agilent Technologies). Sequencing libraries were prepared with the SQK-LSK109 Ligation Sequencing Kit with the following modifications: 48 µl of the sheared DNA (2-2.5 µg) were taken into the end-prep reaction, leaving out the control DNA. The end-prep reaction was changed to an incubation for 30min at 20°C followed by 30min at 65°C followed by a cool down to 4°C in a thermal cycler. The clean-up was performed using AMPure XP beads and 80% ethanol, elution time was changed to 5min. Adapter ligation was extended to an incubation for 60min at room temperature. The ligation mix was then incubated with AMPure XP beads at 0.4x for 10min, clean-up was performed using the Long Fragment Buffer (LFB) and the final library was eluted in a total volume of 31 µl. Library concentrations were measured using the Invitrogen Qubit DNA HS Assay Kit (Q32851, Thermo Fisher Scientific) on a benchtop Quantus fluorometer (Promega). The libraries were loaded (500-600 ng) onto FLO-MIN106 R9.4.1 flow cells with a minimum of 1100 pores available according to the FC Check prior to loading. The flow cells were flushed after around 24 hours for a total of two times per sample with the Flow Cell Wash Kit (EXP-WSH003) per manufacturer's instructions. Sequencing was carried out on a MinION 1B and GridION (Oxford Nanopore Technologies). Three FFPE libraries were prepared, of which DNA for PANEL_A_48 and PANEL_A_49_1 was extracted using the Promega Maxwell RSC Blood DNA Kit and for PANEL_A_49_2 using the QIAamp FFPE Tissue kit.

Adaptive sampling

47 samples (PANEL_A_01-PANEL_A_47) were sequenced on the MinION using ReadFish-enabled targeting on an 8 GB NVIDIA RTX 2080 Ti powered consumer notebook [10]. The targets (Rapid_CNS_A) included regions from the previously published neuropathology panel [12] and CpG sites instrumental in classification by the Heidelberg methylation classifier [6] (available on GitHub

https://github.com/areebapatel/Rapid-CNS2). A 10kb flank was added to the sites on either side to ensure optimal targeting by ReadFish (155 Mb). Guppy 4.2.2's fast basecalling (config dna_r9.4.1_450bps_fast) mode was used to run ReadFish. 12 samples were sequenced using a shorter panel (Rapid_CNS_B: neuropathology gene panel flanked by 10 kb on either side, total 15 Mb) with MinKNOW's in-built adaptive sampling protocol on the GridION. Three samples were sequenced in one run on the GridION. Live basecalling was turned off for all runs.

Variant and copy-number calling

Samples were analyzed using a custom bioinformatics pipeline (areebapatel/Rapid-CNS2.git). FAST5 files were basecalled using ONT' s proprietary software guppy 4.4.1 using the high accuracy configuration. All reads with Q-score >5 were used for analysis. QC and coverage analyses were performed by pycoQC and mosdepth respectively [7, 11]. Adapter trimming by Porechop was followed by minimap2 v2.18 alignment to the hg19 genome, samtools sorting and indexing [8, 9, 17]. SNVs were called using longshot v0.4.1 and PEPPER-Margin-DeepVariant r0.4 [4, 13]. TERT promoter mutations were further validated by mpileup and bcftools. Variant annotation was performed by ANNOVAR [16]. Filtering for clinical relevance was based on the 1000 Genomes (Aug 2015) frequencies and COSMIC 68 database [1, 15]. Copy number plots (100kb bin size) and gene-level copy number files (1kb, 10kb bin sizes) were generated using CNVpytor and a custom script [14].

Methylation-based classification

Methylation calls were made using megalodon v2.3.3 with a guppy v5.0.11 backend [19].
To classify nanopore sequencing derived DNA-methylation profiles of central nervous system tumors, an adapted ad-hoc random forest classifier based on nanoDx was established [5, 6]. It was trained on the publicly available 450k methylation array reference data set of the Heidelberg methylation classifier version 11 (GSE90496) [3]. This data set was preprocessed as described in [3]. For each Nanopore sample, methylation calls overlapping the top 100,000 probes (by mean decrease in accuracy) derived from the Heidelberg methylation classifier were selected. These probes were variance filtered to select the top 10,000 most variable probes. A random forest model with 20,000 trees was trained using ranger [18]. Recalibration was performed by training one vs all generalized linear models for each class. These models were used to obtain a prediction and confidence score for the sample. Ground truth for archival samples was inferred from EPIC array data (from FFPE samples) using the Heidelberg methylation classifier v11b4 predictions.
Methylation families were inferred by aggregating over methylation subclasses from the reference set.

MGMT promoter methylation

Ground truth for MGMT promoter methylation status was inferred from EPIC array analysis and pyrosequencing (6 prospective diagnostic samples). A total of 59 samples (47 Rapid_CNS_A and 12 Rapid_CNS_B samples) were split into 70% training and 30% validation data. In the training data, each of the 212 CpG sites in the MGMT promoter region were subjected to a Student's t-test

to compute the predictive value. 137 sites with p-value <0.01 were selected. The average over these sites was used to train a logistic regression based binomial classifier. The classifier was tested on the validation samples.

Shorter sequencing time and flowcell reuse

Five Rapid_CNS_B libraries (PANEL_B_01, 02, 03, 05, 06) were split based on reads generated in the first 24h (before flushing and reloading) and those generated in the next 48h (after flushing and reloading). These 10 sub-libraries were independently analyzed using the aforementioned pipeline. Since flushing and reloading could also enable loading a new sample, we used this analysis to assess potential for flowcell reuse.

Supplementary results and discussion

Analysis pipeline

The Rapid-CNS[2] pipeline is available as a git repository (https://github.com/areebapatel/Rapid-CNS2). It requires a folder containing FAST5 files or a basecalled FASTQ file as input and generates SNV calls, CNV calls, MGMT promoter status and methylation classification as output (Suppl Fig 7). It can be run on an HPC cluster or a GPU workstation. Basecalling followed by SNV and CNV detection completes within 8 hours (for longshot, 12 hours for PEPPER-Margin-DeepVariant) while methylation calling and classification requires 24 hours depending on amount of data generated. Live methylation calling has the potential to substantially reduce analysis time.

On target rate

Targeted regions showed considerably increased coverage as compared to other regions (Suppl Fig 1a). Within the 47 libraries sequenced using Rapid_CNS_A on the MinION, a clear increase in mean coverage over target regions is shown for all chromosomes (Suppl Fig 1b). Owing to its smaller target size, the 12 libraries sequenced using Rapid_CNS_B displayed higher on-target coverage as compared to those with Rapid_CNS_A. Key genes like IDH1, TERT, BRAF, etc also mirrored the increase in median coverage (Suppl Fig 1c). This could also in part be attributed to the higher sequencing capacity of the GridION.

Mutational analysis

On re-evaluation of samples with discrepant mutations, it was found that Nanopore libraries (PANEL_A_01 and PANEL_A_25) were generated using DNA from frozen sections with infiltration zone and low tumor cell content, whereas the corresponding NGS data was derived from bulk tumor tissue. For PANEL_A_12, the *TERTp* mutation was present in the Nanopore data (detected by mpileup), but was filtered by PEPPER-Margin-DeepVariant and longshot. Owing to major differences in technology (short read NGS vs long read Nanopore-sequencing), variant calling tools (mpileup/Platypus for NGS vs DeepVariant/longshot for Nanopore sequencing) as well as tissue type (FFPE tissue for NGS vs cryoconserved tissue for Nanopore sequencing), we restricted our validation to pathognomonic alterations.

Copy number analysis

Copy number plots obtained using the Rapid-CNS^2 pipeline demonstrated higher resolution and clear visualization of the copy number levels as compared to NGS panel sequencing (Suppl. Fig. 2 (left and centre)). Calculating depth of mapped reads, copy number variations detected were comparable to EPIC array results (Suppl. Fig. 2 (left and right)). Additionally, using smaller bin sizes (1kb, 10kb), genes covered by the copy number variations and their zygosity could also be accurately annotated. Resolution for copy number profiling was maintained across both panels (Rapid_CNS_A and Rapid_CNS_B) (Suppl Fig 2).

Methylation analysis

Owing to non-uniform methylation calling, Bady's MGMT-STP27 method could not be used directly for MGMT promoter assessment [2]. Comparing average methylation over each site in methylated vs unmethylated samples revealed a number of sites had poor predictive value (Suppl. Fig. 3a). Averaging the methylation values over the 137 selected CpG sites showed a clear difference between methylated and unmethylated samples (Suppl. Fig. 3b). The test samples (yellow points) were in concordance with the thresholds proposed by the training data (25% cutoff). The logistic regression classifier trained on the training samples made accurate predictions in all testing samples (72 h runs) (Suppl Fig 3c).

Of the 10,000 probes selected for training in each sample, only ~1400 probes were common in all of the samples (Suppl Fig 3d). The out-of-the bag error for ad-hoc classifiers for each sample was between 0.18-0.20 (Suppl Fig 3e).

Data obtained from a 72h run usually covered >300,000 probes from the 450K array. Loading the entirety of the overlapping probe set from the training set for re-training warranted considerable memory. Methylation classification in Rapid-CNS^2 infers CpG importance from the Heidelberg methylation classifier by selecting sites from the top 100K probes to re-train the ad-hoc classifier. This considerably reduced the time and memory required to perform methylation classification. On average, methylation classification (including I/O processes) took 10 minutes with 16 threads. Complete scores and comparison with EPIC array analysis are reported in the Suppl. Table 2.

Diagnostic samples

Six prospective samples were run using Rapid-CNS^2. As shown in the Suppl. Table 4, five of these were glioblastomas, while one was classified as an IDH mutant, 1p/19q codeleted oligodendroglioma. Histopathological diagnoses for all samples were confirmed by their Rapid-CNS^2 molecular profiling.

Rapid-CNS^2 also accurately profiled PANEL_B_08 as an ATRT-SHH tumor, which was reported as a glioma by histology (Suppl. Fig 4).

Flowcell reuse and shorter sequencing times

Five libraries run using Rapid_CNS_B were split into two each, containing reads generated in the first 24h and those generated in the subsequent 48h after flushing and reloading respectively. Flushing the flowcell depletes all previously loaded sample. We considered data generated after

reloading the flowcell to be equivalent to loading a new library on a previously used flowcell. Since the same sample was loaded again, it avoided any sample or flowcell-related bias. From Suppl. Fig. 5a, each split library contained > 5 million reads. While there was no clear trend observed for the number of reads generated in the first 24h vs next 48h, mean on-target coverage for all libraries was similar to that observed with Rapid_CNS_A libraries sequenced for 72h (Suppl. Fig. 6c, 1a). As shown in Suppl. Fig. 6b and d, complete concordance was reported for all pathognomonic alterations (*IDH1, TERTp, MGMT* promoter methylation and copy number alterations). Methylation families were correctly identified in all split libraries, 4 of which also reported the accurate methylation sub-class as identified by the corresponding EPIC array-based classification (same as their corresponding 72h runs, Suppl. file). Copy number profiles were identical for the complete 72h run, first 24h run and next 48h run for all split libraries (Suppl. Fig. 6d). For a 24h run, sequencing with the shorter panel (Rapid_CNS_B) resulted in mean on-target coverage equivalent to a 72h run with the longer panel (Rapid_CNS_A). This demonstrated that sequencing time can be reduced proportionally by decreasing target sizes. As flowcell quality is maintained, subsequent flushing and reloading would enable effective reuse with multiple samples.

FFPE samples

Three libraries were run with two FFPE samples (PANEL_A_48 (72h), PANEL_A_49_1 and PANEL_A_49_2 (stopped running at 24h due to poor sequencing)) to assess feasibility of the approach. As evident from Suppl. Fig 7 all FFPE runs displayed very low read counts (140K -160K reads) as compared to a cryopreserved sample (~10M reads). Channel activity was also poor. While flushing after 24h intervals in PANEL_A_48 increased channel activity and read count, it did not generate sufficient data for analysis. This could be potentially due to low fragment lengths in FFPE samples. Thus, in its current state, Rapid-CNS^2 is restricted to use with cryopreserved or fresh CNS tumor samples. However, the potential for further advances in Nanopore technology cannot be underestimated, and FFPE sample processing may be feasible in the near future.

Costs involved:

Currently, a complete molecular diagnostic workup including NGS panel sequencing and EPIC array analysis costs about 410 EUR per sample. This seemingly lower cost is offset by the initial investment which could amount to > 300,000 EUR, and the need to have sufficient case numbers to efficiently run EPIC or NGS. In comparison, MinION costs about 1,000 EUR and the GridION (5 samples) costs about 50,000 EUR. For Rapid-CNS^2 runs, flowcell costs currently (potentially depending on market) start from 430 EUR per flowcell (96-pack) to 810 EUR (single flowcell). Library preparation and two flowcell washes then amount to 120 EUR. Thus, starting cost per sample for Rapid-CNS^2 adds up to 550 EUR (excluding electricity and other auxiliary costs). The flowcell cost is the limiting factor in the total assay costs. However, as a cost-efficient solution flowcells can be reused, reducing the total cost of Rapid-CNS^2 to 315 EUR per sample and thus making it competitive in comparison to conventional methods. Reducing panel size could facilitate multiple reuses of the flowcell, thus reducing costs even further.

References:

1. Auton A, Abecasis GR, Altshuler DM, Durbin RM, Abecasis GR, Bentley DR, Chakravarti A, Clark AG, Donnelly P, Eichler EE, Flicek P, Gabriel SB, Gibbs RA, Green ED, Hurles ME, Knoppers BM, Korbel JO, Lander ES, Lee C, Lehrach H, Mardis ER, Marth GT, McVean GA, Nickerson DA, Schmidt JP, Sherry ST, Wang J, Wilson RK, Gibbs RA, Boerwinkle E, Doddapaneni H, Han Y, Korchina V, Kovar C, Lee S, Muzny D, Reid JG, Zhu Y, Wang J, Chang Y, Feng Q, Fang X, Guo X, Jian M, Jiang H, Jin X, Lan T, Li G, Li J, Li Y, Liu S, Liu X, Lu Y, Ma X, Tang M, Wang B, Wang G, Wu H, Wu R, Xu X, Yin Y, Zhang D, Zhang W, Zhao J, Zhao M, Zheng X, Lander ES, Altshuler DM, Gabriel SB, Gupta N, Gharani N, Toji LH, Gerry NP, Resch AM, Flicek P, Barker J, Clarke L, Gil L, Hunt SE, Kelman G, Kulesha E, Leinonen R, McLaren WM, Radhakrishnan R, Roa A, Smirnov D, Smith RE, Streeter I, Thormann A, Toneva I, Vaughan B, Zheng-Bradley X, Bentley DR, Grocock R, Humphray S, James T, Kingsbury Z, Lehrach H, Sudbrak R, Albrecht MW, Amstislavskiy VS, Borodina TA, Lienhard M, Mertes F, Sultan M, Timmermann B, Yaspo M-L, Mardis ER, Wilson RK, Fulton L, Fulton R, Sherry ST, Ananiev V, Belaia Z, Beloslyudtsev D, Bouk N, Chen C, Church D, Cohen R, Cook C, Garner J, Hefferon T, Kimelman M, Liu C, Lopez J, Meric P, O'Sullivan C, Ostapchuk Y, Phan L, Ponomarov S, Schneider V, Shekhtman E, Sirotkin K, Slotta D, Zhang H, McVean GA, Durbin RM, Balasubramaniam S, Burton J, Danecek P, Keane TM, Kolb-Kokocinski A, McCarthy S, Stalker J, Quail M, Schmidt JP, Davies CJ, Gollub J, Webster T, Wong B, Zhan Y, Auton A, Campbell CL, Kong Y, Marcketta A, Gibbs RA, Yu F, Antunes L, Bainbridge M, Muzny D, Sabo A, Huang Z, Wang J, Coin LJM, Fang L, Guo X, Jin X, Li G, Li Q, Li Y, Li Z, Lin H, Liu B, Luo R, Shao H, Xie Y, Ye C, Yu C, Zhang F, Zheng H, Zhu H, Alkan C, Dal E, Kahveci F, Marth GT, Garrison EP, Kural D, Lee W-P, Fung Leong W, Stromberg M, Ward AN, Wu J, Zhang M, Daly MJ, DePristo MA, Handsaker RE, Altshuler DM, Banks E, Bhatia G, del Angel G, Gabriel SB, Genovese G, Gupta N, Li H, Kashin S, Lander ES, McCarroll SA, Nemesh JC, Poplin RE, Yoon SC, Lihm J, Makarov V, Clark AG, Gottipati S, Keinan A, Rodriguez-Flores JL, Korbel JO, Rausch T, Fritz MH, Stütz AM, Flicek P, Beal K, Clarke L, Datta A, Herrero J, McLaren WM, Ritchie GRS, Smith RE, Zerbino D, Zheng-Bradley X, Sabeti PC, Shlyakhter I, Schaffner SF, Vitti J, Cooper DN, Ball E v, Stenson PD, Bentley DR, Barnes B, Bauer M, Keira Cheetham R, Cox A, Eberle M, Humphray S, Kahn S, Murray L, Peden J, Shaw R, Kenny EE, Batzer MA, Konkel MK, Walker JA, MacArthur DG, Lek M, Sudbrak R, Amstislavskiy VS, Herwig R, Mardis ER, Ding L, Koboldt DC, Larson D, Ye K, Gravel S, Consortium T 1000 GP, authors C, committee S, group P, Medicine BC of, BGI-Shenzhen, Harvard BI of MIT and, Research CI for M, European Molecular Biology Laboratory EBI, Illumina, Genetics MPI for M, University MGI at W, Health USNI of, Oxford U of, Institute WTS, group A, Affymetrix, Medicine AEC of, University B, College B, Laboratory CSH, University C, Laboratory EMB, University H, Database HGM, Sinai IS of M at M, University LS, Hospital MG, University M, National Eye Institute NIH (2015) A global reference for human genetic variation. Nature 526:68–74. doi: 10.1038/nature15393
2. Bady P, Sciuscio D, Diserens A-C, Bloch J, van den Bent MJ, Marosi C, Dietrich P-Y, Weller M, Mariani L, Heppner FL, Mcdonald DR, Lacombe D, Stupp R, Delorenzi M, Hegi ME (2012) MGMT methylation analysis of glioblastoma on the Infinium methylation BeadChip identifies two distinct CpG regions associated with gene silencing and outcome, yielding a prediction model for comparisons across datasets, tumor grades, and CIMP-status. Acta neuropathologica 124:547–560. doi: 10.1007/s00401-012-1016-2
3. Capper D, Jones DTW, Sill M, Hovestadt V, Schrimpf D, Sturm D, Koelsche C, Sahm F, Chavez L, Reuss DE, Kratz A, Wefers AK, Huang K, Pajtler KW, Schweizer L, Stichel D, Olar A, Engel NW, Lindenberg K, Harter PN, Braczynski AK, Plate KH, Dohmen H, Garvalov BK, Coras R, Hölsken A, Hewer E, Bewerunge-Hudler M, Schick M, Fischer R, Beschorner R, Schittenhelm J, Staszewski O, Wani K, Varlet P, Pages M, Temming P, Lohmann D, Selt F, Witt H, Milde T, Witt O, Aronica E, Giangaspero F, Rushing E, Scheurlen W, Geisenberger C, Rodriguez FJ, Becker A, Preusser M, Haberler C, Bjerkvig R, Cryan J, Farrell M, Deckert M, Hench J, Frank S, Serrano J, Kannan K, Tsirigos A, Brück W, Hofer S, Brehmer S, Seiz-Rosenhagen M, Hänggi D, Hans V, Rozsnoki S, Hansford JR, Kohlhof P, Kristensen BW, Lechner M, Lopes B, Mawrin C, Ketter R, Kulozik A, Khatib Z, Heppner F, Koch A, Jouvet A, Keohane C, Mühleisen H, Mueller W, Pohl U, Prinz M, Benner A, Zapatka M, Gottardo NG, Driever PH, Kramm CM, Müller HL, Rutkowski S, von Hoff K, Frühwald MC, Gnekow A, Fleischhack G, Tippelt S, Calaminus G, Monoranu C-M, Perry A, Jones C, Jacques TS, Radlwimmer B, Gessi M, Pietsch T, Schramm J, Schackert G, Westphal M, Reifenberger G, Wesseling P, Weller M, Collins VP, Blümcke I, Bendszus M, Debus J, Huang A, Jabado N, Northcott PA, Paulus W, Gajjar A, Robinson GW, Taylor MD, Jaunmuktane Z, Ryzhova M, Platten M, Unterberg A, Wick W, Karajannis MA, Mittelbronn M, Acker T, Hartmann C, Aldape K, Schüller U, Buslei R, Lichter P, Kool M, Herold-Mende C, Ellison

DW, Hasselblatt M, Snuderl M, Brandner S, Korshunov A, von Deimling A, Pfister SM (2018) DNA methylation-based classification of central nervous system tumours. Nature 555:469–474. doi: 10.1038/nature26000
4. Edge P, Bansal V (2019) Longshot enables accurate variant calling in diploid genomes from single-molecule long read sequencing. Nature Communications 10:4660. doi: 10.1038/s41467-019-12493-y
5. Euskirchen P, Bielle F, Labreche K, Kloosterman WP, Rosenberg S, Daniau M, Schmitt C, Masliah-Planchon J, Bourdeaut F, Dehais C, Marie Y, Delattre J-Y, Idbaih A (2017) Same-day genomic and epigenomic diagnosis of brain tumors using real-time nanopore sequencing. Acta neuropathologica 134:691–703. doi: 10.1007/s00401-017-1743-5
6. Kuschel LP, Hench J, Frank S, Hench IB, Girard E, Blanluet M, Masliah-Planchon J, Misch M, Onken J, Czabanka M, Karau P, Ishaque N, Hain EG, Heppner F, Idbaih A, Behr N, Harms C, Capper D, Euskirchen P (2021) Robust methylation-based classification of brain tumors using nanopore sequencing. medRxiv 2021.03.06.21252627. doi: 10.1101/2021.03.06.21252627
7. Leger A, Leonardi T (2019) pycoQC, interactive quality control for Oxford Nanopore Sequencing. Journal of Open Source Software 4:1236. doi: 10.21105/joss.01236
8. Li H (2018) Minimap2: pairwise alignment for nucleotide sequences. Bioinformatics 34:3094–3100. doi: 10.1093/bioinformatics/bty191
9. Li H, Handsaker B, Wysoker A, Fennell T, Ruan J, Homer N, Marth G, Abecasis G, Durbin R, 1000 Genome Project Data Processing Subgroup (2009) The Sequence Alignment/Map format and SAMtools. Bioinformatics 25:2078–2079. doi: 10.1093/bioinformatics/btp352
10. Payne A, Holmes N, Clarke T, Munro R, Debebe BJ, Loose M (2021) Readfish enables targeted nanopore sequencing of gigabase-sized genomes. Nature Biotechnology 39:442–450. doi: 10.1038/s41587-020-00746-x
11. Pedersen BS, Quinlan AR (2018) Mosdepth: quick coverage calculation for genomes and exomes. Bioinformatics 34:867–868. doi: 10.1093/bioinformatics/btx699
12. Sahm F, Schrimpf D, Jones DTW, Meyer J, Kratz A, Reuss D, Capper D, Koelsche C, Korshunov A, Wiestler B, Buchhalter I, Milde T, Selt F, Sturm D, Kool M, Hummel M, Bewerunge-Hudler M, Mawrin C, Schüller U, Jungk C, Wick A, Witt O, Platten M, Herold-Mende C, Unterberg A, Pfister SM, Wick W, von Deimling A (2016) Next-generation sequencing in routine brain tumor diagnostics enables an integrated diagnosis and identifies actionable targets. Acta Neuropathologica 131:903–910. doi: 10.1007/s00401-015-1519-8
13. Shafin K, Pesout T, Chang P-C, Nattestad M, Kolesnikov A, Goel S, Baid G, Eizenga JM, Miga KH, Carnevali P, Jain M, Carroll A, Paten B (2021) Haplotype-aware variant calling enables high accuracy in nanopore long-reads using deep neural networks. bioRxiv 2021.03.04.433952. doi: 10.1101/2021.03.04.433952
14. Suvakov M, Panda A, Diesh C, Holmes I, Abyzov A (2021) CNVpytor: a tool for CNV/CNA detection and analysis from read depth and allele imbalance in whole genome sequencing. bioRxiv 2021.01.27.428472. doi: 10.1101/2021.01.27.428472
15. Tate JG, Bamford S, Jubb HC, Sondka Z, Beare DM, Bindal N, Boutselakis H, Cole CG, Creatore C, Dawson E, Fish P, Harsha B, Hathaway C, Jupe SC, Kok CY, Noble K, Ponting L, Ramshaw CC, Rye CE, Speedy HE, Stefancsik R, Thompson SL, Wang S, Ward S, Campbell PJ, Forbes SA (2019) COSMIC: the Catalogue Of Somatic Mutations In Cancer. Nucleic acids research 47:D941–D947. doi: 10.1093/nar/gky1015
16. Wang K, Li M, Hakonarson H (2010) ANNOVAR: functional annotation of genetic variants from high-throughput sequencing data. Nucleic acids research 38:e164. doi: 10.1093/nar/gkq603
17. Wick RR, Judd LM, Gorrie CL, Holt KE (2017) Completing bacterial genome assemblies with multiplex MinION sequencing. Microbial Genomics 3. doi: 10.1099/mgen.0.000132
18. Wright MN, Ziegler A (2017) **ranger**: A Fast Implementation of Random Forests for High Dimensional Data in *C++* and *R*. Journal of Statistical Software 77. doi: 10.18637/jss.v077.i01
19. nanoporetech/megalodon: Megalodon is a research command line tool to extract high accuracy modified base and sequence variant calls from raw nanopore reads by anchoring the information rich basecalling neural network output to a reference genome/transriptome. https://github.com/nanoporetech/megalodon. Accessed 2 Dec 2020

Library	Platform	Panel	Matching_conventional_data	In Fig 1	Histological diagnosis	Final type/subtype diagnosis	Methylation classification
PANEL_A_01	MinION	Rapid_CNS_A	Yes	Yes	diffuse glioma	Glioblastoma (IDH wildtype)	GBM_MES
PANEL_A_02	MinION	Rapid_CNS_A	Yes	Yes	diffuse glioma	Glioblastoma (IDH wildtype)	GBM_RTK_II
PANEL_A_03	MinION	Rapid_CNS_A	Yes	Yes	diffuse glioma	Glioblastoma (IDH wildtype)	GBM_RTK_II
PANEL_A_04	MinION	Rapid_CNS_A	Yes	Yes	diffuse glioma	Glioblastoma (IDH wildtype)	GBM_RTK_II
PANEL_A_05	MinION	Rapid_CNS_A	Yes	Yes	diffuse glioma	Glioblastoma (IDH wildtype)	GBM_RTK_II
PANEL_A_06	MinION	Rapid_CNS_A	Yes	Yes	diffuse glioma	Glioblastoma (IDH wildtype)	GBM_RTK_II
PANEL_A_07	MinION	Rapid_CNS_A	Yes	Yes	diffuse glioma	Glioblastoma (IDH wildtype)	GBM_RTK_II
PANEL_A_08	MinION	Rapid_CNS_A	Yes	Yes	diffuse glioma	Glioblastoma (IDH wildtype)	GBM_RTK_II
PANEL_A_09	MinION	Rapid_CNS_A	Yes	Yes	diffuse glioma	Glioblastoma (IDH wildtype)	GBM_RTK_II
PANEL_A_10	MinION	Rapid_CNS_A	Yes	Yes	diffuse glioma	Glioblastoma (IDH wildtype)	GBM_RTK_II
PANEL_A_11	MinION	Rapid_CNS_A	Yes	Yes	diffuse glioma	Glioblastoma (IDH wildtype)	GBM_RTK_I
PANEL_A_12	MinION	Rapid_CNS_A	Yes	Yes	diffuse glioma	Glioblastoma (IDH wildtype)	GBM_RTK_II
PANEL_A_13	MinION	Rapid_CNS_A	Yes	Yes	diffuse glioma	Glioblastoma (IDH wildtype)	GBM_RTK_II
PANEL_A_14	MinION	Rapid_CNS_A	Yes	Yes	diffuse glioma	Glioblastoma (IDH wildtype)	GBM_RTK_II
PANEL_A_15	MinION	Rapid_CNS_A	Yes	Yes	diffuse glioma	Glioblastoma (IDH wildtype)	GBM_RTK_II
PANEL_A_16	MinION	Rapid_CNS_A	Yes	Yes	diffuse glioma	Astrocytoma (IDH mutant)	A_IDH_HG
PANEL_A_17	MinION	Rapid_CNS_A	Yes	Yes	diffuse glioma	Oligodendroglioma (IDH mutant)	O_IDH
PANEL_A_18	MinION	Rapid_CNS_A	Yes	Yes	diffuse glioma	Oligodendroglioma (IDH mutant)	O_IDH
PANEL_A_19	MinION	Rapid_CNS_A	Yes	Yes	diffuse glioma	Astrocytoma (IDH mutant)	A_IDH_HG
PANEL_A_20	MinION	Rapid_CNS_A	Yes	Yes	diffuse glioma	Astrocytoma (IDH mutant)	A_IDH_HG
PANEL_A_21	MinION	Rapid_CNS_A	Yes	Yes	diffuse glioma	Astrocytoma (IDH mutant)	A_IDH
PANEL_A_22	MinION	Rapid_CNS_A	Yes	Yes	diffuse glioma	Oligodendroglioma (IDH mutant)	O_IDH
PANEL_A_23	MinION	Rapid_CNS_A	Yes	Yes	diffuse glioma	Astrocytoma (IDH mutant)	A_IDH_HG
PANEL_A_24	MinION	Rapid_CNS_A	Yes	Yes	diffuse glioma	Astrocytoma (IDH mutant)	A_IDH
PANEL_A_25	MinION	Rapid_CNS_A	Yes	Yes	diffuse glioma	Astrocytoma	A_IDH
PANEL_A_26	MinION	Rapid_CNS_A	Yes	Yes	diffuse glioma	Oligodendroglioma (IDH mutant)	O_IDH
PANEL_A_27	MinION	Rapid_CNS_A	Yes	Yes	diffuse glioma	Astrocytoma (IDH mutant)	A_IDH_HG
PANEL_A_28	MinION	Rapid_CNS_A	Yes	Yes	diffuse glioma	Glioblastoma (IDH wildtype)	GBM_RTK_II
PANEL_A_29	MinION	Rapid_CNS_A	Yes	Yes	diffuse glioma	Glioblastoma (IDH wildtype)	GBM_RTK_II
PANEL_A_30	MinION	Rapid_CNS_A	Yes	Yes	diffuse glioma	Glioblastoma (IDH wildtype)	GBM_RTK_II
PANEL_A_31	MinION	Rapid_CNS_A	Yes	Yes	diffuse glioma	Oligodendroglioma (IDH mutant)	O_IDH
PANEL_A_32	MinION	Rapid_CNS_A	Yes	Yes	diffuse glioma	Glioblastoma (IDH wildtype)	GBM_RTK_II
PANEL_A_33	MinION	Rapid_CNS_A	Yes	Yes	diffuse glioma	Glioblastoma (IDH wildtype)	GBM_RTK_II
PANEL_A_34	MinION	Rapid_CNS_A	Yes	Yes	diffuse glioma	Glioblastoma (IDH wildtype)	GBM_RTK_II
PANEL_A_35	MinION	Rapid_CNS_A	Yes	Yes	diffuse glioma	Glioblastoma (IDH wildtype)	GBM_RTK_II
PANEL_A_36	MinION	Rapid_CNS_A	Yes	Yes	diffuse glioma	Glioblastoma (IDH wildtype)	GBM_RTK_II
PANEL_A_37	MinION	Rapid_CNS_A	Yes	Yes	low-grade glioma	Pilocytic astrocyoma	LGG_PA_PF
PANEL_A_38	MinION	Rapid_CNS_A	Yes	Yes	low-grade glioma	Pilocytic astrocyoma	LGG_PA_MID
PANEL_A_39	MinION	Rapid_CNS_A	Yes	Yes	low-grade glioma	Pilocytic astrocyoma	LGG_PA_PF
PANEL_A_40	MinION	Rapid_CNS_A	Yes	Yes	low-grade glioma	Pilocytic astrocyoma	LGG_PA_PF
PANEL_A_41	MinION	Rapid_CNS_A	Yes	Yes	diffuse glioma	Glioblastoma (IDH wildtype)	GBM_RTK_II
PANEL_A_42	MinION	Rapid_CNS_A	Yes	Yes	diffuse glioma	Glioblastoma (IDH wildtype)	GBM_RTK_II
PANEL_A_43	MinION	Rapid_CNS_A	Yes	Yes	diffuse glioma	Glioblastoma (IDH wildtype)	GBM_RTK_II
PANEL_A_44	MinION	Rapid_CNS_A	Yes	Yes	diffuse glioma	Glioblastoma (IDH wildtype)	GBM_RTK_II
PANEL_A_45	MinION	Rapid_CNS_A	Yes	Yes	diffuse glioma	Glioblastoma (IDH wildtype)	GBM_RTK_II
PANEL_A_46	MinION	Rapid_CNS_A	No	No (prospective diagnostic sample)	diffuse glioma	Glioblastoma (IDH wildtype)	GBM_RTK_II
PANEL_A_47	MinION	Rapid_CNS_A	No	No (prospective diagnostic sample)	diffuse glioma	Glioblastoma (IDH wildtype)	GBM_MES
PANEL_B_01	GridION	Rapid_CNS_B	Yes	Yes	diffuse glioma	Glioblastoma (IDH wildtype)	GBM_RTK_II
PANEL_B_02	GridION	Rapid_CNS_B	Yes	Yes	diffuse glioma	Glioblastoma (IDH wildtype)	GBM_RTK_II
PANEL_B_03	GridION	Rapid_CNS_B	Yes	Yes	diffuse glioma	Glioblastoma (IDH wildtype)	GBM_RTK_II
PANEL_B_04	GridION	Rapid_CNS_B	Yes	Yes	diffuse glioma	Glioblastoma (IDH wildtype)	GBM_RTK_II
PANEL_B_05	GridION	Rapid_CNS_B	Yes	Yes	diffuse glioma	Glioblastoma (IDH wildtype)	GBM_RTK_II
PANEL_B_06	GridION	Rapid_CNS_B	Yes	Yes	diffuse glioma	Glioblastoma (IDH wildtype)	GBM_RTK_II
PANEL_B_07	GridION	Rapid_CNS_B	Yes	Yes	diffuse glioma	Glioblastoma (IDH wildtype)	GBM_RTK_II
PANEL_B_08	GridION	Rapid_CNS_B	Yes	No (AT/RT final diagnosis)	diffuse glioma	Atypical teratoid, rhabdoid tumor	ATRT_SHH
PANEL_B_09	GridION	Rapid_CNS_B	No	No (prospective diagnostic sample)	diffuse glioma	Oligodendroglioma (IDH mutant)	O_IDH
PANEL_B_10	GridION	Rapid_CNS_B	No	No (prospective diagnostic sample)	diffuse glioma	Glioblastoma (IDH wildtype)	GBM_RTK_II
PANEL_B_11	GridION	Rapid_CNS_B	No	No (prospective diagnostic sample)	diffuse glioma	Glioblastoma (IDH wildtype)	GBM_RTK_II
PANEL_B_12	GridION	Rapid_CNS_B	No	No (prospective diagnostic sample)	diffuse glioma	Glioblastoma (IDH wildtype)	GBM_RTK_II

Suppl. Table 1: Overview of all samples in this study

Library	Number of features	Calibrated score (RF)	Predicted class (RF)	Calibrated confidence score (GLM)	Predicted class (GLM)	Predicted class (EPIC)	Concordance
PANEL_A_01	10000	0,011159664	GBM_MES	6,540558417	GBM_MES	GBM_MES	Sub-class
PANEL_A_02	10000	0,996246535	GBM_RTK_II	99,62465346	GBM_RTK_II	GBM_RTK_II	Sub-class
PANEL_A_03	10000	0,999999998	GBM_RTK_II	99,99999982	GBM_RTK_II	GBM_RTK_I	Family
PANEL_A_04	10000	0,004540359	GBM_MES	63,50232342	GBM_RTK_II	GBM_RTK_I	Family
PANEL_A_05	10000	0,008446298	GBM_RTK_I	94,63140789	GBM_RTK_II	GBM_RTK_II	Sub-class
PANEL_A_06	10000	0,999999991	GBM_RTK_II	99,99999908	GBM_RTK_II	GBM_RTK_II	Sub-class
PANEL_A_07	10000	0,003829409	GBM_MES	77,21341357	GBM_RTK_II	GBM_RTK_II	Sub-class
PANEL_A_08	10000	0,997740399	GBM_RTK_II	99,77403994	GBM_RTK_II	GBM_MES	Family
PANEL_A_09	10000	0,907490998	GBM_RTK_II	90,74909981	GBM_RTK_II	GBM_RTK_II	Sub-class
PANEL_A_10	10000	0,000975314	GBM_RTK_I	96,05291628	GBM_RTK_II	GBM_RTK_I	Family
PANEL_A_11	10000	0,997461032	GBM_RTK_I	99,74610323	GBM_RTK_I	GBM_RTK_I	Sub-class
PANEL_A_12	10000	0,004372027	GBM_MES	13,01771473	GBM_RTK_II	GBM_RTK_II	Sub-class
PANEL_A_13	10000	0,000409539	GBM_RTK_I	99,74301447	GBM_RTK_II	GBM_RTK_I	Family
PANEL_A_14	10000	0,99039392	GBM_RTK_II	99,039392	GBM_RTK_II	GBM_RTK_II	Sub-class
PANEL_A_15	10000	0,914692029	GBM_RTK_II	91,46920291	GBM_RTK_II	GBM_RTK_II	Sub-class
PANEL_A_16	10000	0,542949575	A_IDH_HG	54,29495747	A_IDH_HG	A_IDH	Family
PANEL_A_17	10000	0,995503384	O_IDH	99,55033845	O_IDH	O_IDH	Sub-class
PANEL_A_18	10000	0,999973347	O_IDH	99,99733469	O_IDH	O_IDH	Sub-class
PANEL_A_19	10000	0,524944644	A_IDH_HG	52,4944644	A_IDH_HG	A_IDH_HG	Sub-class
PANEL_A_20	10000	0,945875372	A_IDH_HG	94,58753725	A_IDH_HG	A_IDH_HG	Sub-class
PANEL_A_21	10000	0,999976579	A_IDH	99,99765791	A_IDH	A_IDH	Sub-class
PANEL_A_22	10000	0,999969496	O_IDH	99,99694963	O_IDH	O_IDH	Sub-class
PANEL_A_23	10000	0,894935069	A_IDH_HG	89,49350689	A_IDH_HG	A_IDH_HG	Sub-class
PANEL_A_24	10000	0,999135784	A_IDH	99,9135784	A_IDH	A_IDH	Sub-class
PANEL_A_25	10000	0,915488785	A_IDH	91,54887846	A_IDH	A_IDH	Sub-class
PANEL_A_26	10000	0,997204211	O_IDH	99,72042108	O_IDH	O_IDH	Sub-class
PANEL_A_27	10000	0,994714004	A_IDH_HG	99,47140038	A_IDH_HG	A_IDH_HG	Sub-class
PANEL_A_28	10000	0,999777797	GBM_RTK_II	99,97777967	GBM_RTK_II	GBM_RTK_II	Sub-class
PANEL_A_29	10000	0,001740446	GBM_RTK_I	95,14750202	GBM_RTK_II	GBM_RTK_I	Family
PANEL_A_30	10000	0,999999998	GBM_RTK_II	99,99999983	GBM_RTK_II	GBM_RTK_II	Sub-class
PANEL_A_31	10000	0,997517909	O_IDH	99,75179086	O_IDH	O_IDH	Sub-class
PANEL_A_32	10000	0,003847069	GBM_RTK_I	99,61328256	GBM_RTK_II	GBM_RTK_I	Family
PANEL_A_33	10000	0,997998384	GBM_RTK_II	99,79983843	GBM_RTK_II	GBM_RTK_II	Sub-class
PANEL_A_34	10000	0,247060925	GBM_RTK_I	36,76086648	GBM_RTK_II	GBM_RTK_I	Family
PANEL_A_35	10000	0,002270122	GBM_MES	97,20798679	GBM_RTK_II	GBM_RTK_II	Sub-class
PANEL_A_36	10000	0,999999997	GBM_RTK_II	99,99999967	GBM_RTK_II	GBM_RTK_II	Sub-class
PANEL_A_37	10000	0,982323764	LGG_PA_PF	98,23237639	LGG_PA_PF	LGG_PA_PF	Sub-class
PANEL_A_38	10000	0,662668227	LGG_PA_MID	66,26682267	LGG_PA_MID	LGG_PA_MID	Sub-class
PANEL_A_39	10000	0,994297964	LGG_PA_PF	99,42979644	LGG_PA_PF	LGG_PA_PF	Sub-class
PANEL_A_40	10000	0,929645857	LGG_PA_PF	92,9645857	LGG_PA_PF	LGG_PA_PF	Sub-class
PANEL_A_41	10000	0,999999994	GBM_RTK_II	99,99999944	GBM_RTK_II	GBM_RTK_II	Sub-class
PANEL_A_42	10000	0,001996373	GBM_MES	94,7288772	GBM_RTK_II	GBM_RTK_II	Sub-class
PANEL_A_43	10000	0,996190259	GBM_RTK_II	99,61902589	GBM_RTK_II	GBM_RTK_II	Sub-class
PANEL_A_44	10000	0,984196525	GBM_RTK_II	98,41965254	GBM_RTK_II	GBM_RTK_II	Sub-class
PANEL_A_45	10000	0,009189499	GBM_MES	19,26935908	GBM_RTK_II	GBM_RTK_II	Sub-class
PANEL_A_46	10000	0,999994905	GBM_RTK_II	99,99949049	GBM_RTK_II		
PANEL_A_47	10000	0,016392181	GBM_MES	11,9140475	GBM_MES		
PANEL_B_01	10000	0,008067548	GBM_MES	7,596287338	GBM_RTK_II	GBM_MES	Family
PANEL_B_02	10000	0,995937632	GBM_RTK_II	99,59376318	GBM_RTK_II	GBM_RTK_II	Sub-class
PANEL_B_03	10000	0,755716876	GBM_RTK_II	75,57168763	GBM_RTK_II	GBM_RTK_I	Family
PANEL_B_04	10000	0,999646153	GBM_RTK_II	99,96461527	GBM_RTK_II	GBM_RTK_II	Sub-class
PANEL_B_05	10000	0,032874718	GBM_RTK_I	80,94821492	GBM_RTK_II	GBM_MES	Family
PANEL_B_06	10000	0,999940639	GBM_RTK_II	99,99406395	GBM_RTK_II	GBM_RTK_II	Sub-class
PANEL_B_07	10000	0,004206767	GBM_MES	9,747388487	GBM_RTK_II	GBM_MES	Family
PANEL_B_08	10000	0,144500592	ATRT_SHH	14,45005921	ATRT_SHH	ATRT_SHH	Sub-class
PANEL_B_09	10000	0,952473286	O_IDH	95,24732858	O_IDH		
PANEL_B_10	10000	0,001720594	GBM_RTK_I	99,25069646	GBM_RTK_II		
PANEL_B_11	10000	0,003673239	GBM_RTK_I	16,16365579	GBM_RTK_II		
PANEL_B_12	10000	0,023254435	GBM_RTK_I	74,71735342	GBM_RTK_II		
PANEL_B_01_1	10000	0,006593588	GBM_MES	11,52569581	GBM_RTK_II	GBM_MES	Family
PANEL_B_01_2	10000	0,006453965	GBM_MES	5,921099306	GBM_RTK_II	GBM_MES	Family
PANEL_B_02_1	10000	0,003143455	GBM_RTK_I	72,39732753	GBM_RTK_II	GBM_RTK_II	Sub-class
PANEL_B_02_2	10000	0,986512444	GBM_RTK_II	98,65124435	GBM_RTK_II	GBM_RTK_II	Sub-class
PANEL_B_03_1	10000	0,002986742	GBM_RTK_I	73,68474891	GBM_RTK_II	GBM_RTK_I	Family
PANEL_B_03_2	10000	0,002766173	GBM_RTK_I	49,69252738	GBM_RTK_II	GBM_RTK_I	Family
PANEL_B_05_1	10000	0,079277925	GBM_RTK_I	41,81810666	GBM_RTK_II	GBM_MES	Family
PANEL_B_05_2	10000	0,098051325	GBM_RTK_I	9,805132547	GBM_RTK_I	GBM_MES	Family
PANEL_B_06_1	10000	0,999285339	GBM_RTK_II	99,92853386	GBM_RTK_II	GBM_RTK_II	Sub-class
PANEL_B_06_2	10000	0,999803912	GBM_RTK_II	99,98039121	GBM_RTK_II	GBM_RTK_II	Sub-class

Suppl. Table 2. Methylation classification results and concordance with EPIC array

Library	Average	Prediction probability	Predicted	Truth
PANEL_A_01	14,23577	0	Unmethylated	Unmethylated
PANEL_A_02	17,76058	0	Unmethylated	Unmethylated
PANEL_A_03	24,27226	0	Unmethylated	Unmethylated
PANEL_A_04	11,78686	0	Unmethylated	Unmethylated
PANEL_A_05	11,25839	0	Unmethylated	Unmethylated
PANEL_A_06	4,353285	0	Unmethylated	Unmethylated
PANEL_A_07	10,18175	0	Unmethylated	Unmethylated
PANEL_A_08	12,26058	0	Unmethylated	Unmethylated
PANEL_A_09	8,331387	0	Unmethylated	Unmethylated
PANEL_A_10	10,03431	0	Unmethylated	Unmethylated
PANEL_A_11	13,38394	0	Unmethylated	Unmethylated
PANEL_A_12	17,20438	0	Unmethylated	Unmethylated
PANEL_A_13	11,04672	0	Unmethylated	Unmethylated
PANEL_A_14	11,24964	0	Unmethylated	Unmethylated
PANEL_A_15	12,46715	0	Unmethylated	Unmethylated
PANEL_A_16	46,67372	1	Methylated	Methylated
PANEL_A_17	65,74599	1	Methylated	Methylated
PANEL_A_18	54,38832	1	Methylated	Methylated
PANEL_A_19	40,55985	1	Methylated	Methylated
PANEL_A_20	17,57455	0	Unmethylated	Unmethylated
PANEL_A_21	29,44161	0,999999999	Methylated	Methylated
PANEL_A_22	56,83504	1	Methylated	Methylated
PANEL_A_23	48,49781	1	Methylated	Methylated
PANEL_A_24	37,72044	1	Methylated	Methylated
PANEL_A_25	43,43066	1	Methylated	Methylated
PANEL_A_26	40,71825	1	Methylated	Methylated
PANEL_A_27	31,57372	1	Methylated	Methylated
PANEL_A_28	17,96563	0	Unmethylated	Unmethylated
PANEL_A_29	18,47518	0	Unmethylated	Unmethylated
PANEL_A_30	44,16204	1	Methylated	Methylated
PANEL_A_31	39,70876	1	Methylated	Methylated
PANEL_A_32	57,55328	1	Methylated	Methylated
PANEL_A_33	18,96552	0	Unmethylated	Unmethylated
PANEL_A_34	17,48175	0	Unmethylated	Unmethylated
PANEL_A_35	13,56204	0	Unmethylated	Unmethylated
PANEL_A_36	74,94161	1	Methylated	Methylated
PANEL_A_37	21,08321	0	Unmethylated	Unmethylated
PANEL_A_38	11,84307	0	Unmethylated	Unmethylated
PANEL_A_39	15,47445	0	Unmethylated	Unmethylated
PANEL_A_40	10,59124	0	Unmethylated	Unmethylated
PANEL_A_41	8,029197	0	Unmethylated	Unmethylated
PANEL_A_42	6,334307	0	Unmethylated	Unmethylated
PANEL_A_43	9,086861	0	Unmethylated	Unmethylated
PANEL_A_44	20,80146	0	Unmethylated	Unmethylated
PANEL_A_45	60,65182	1	Methylated	Methylated
PANEL_A_46	8,710219	0	Unmethylated	Unmethylated
PANEL_A_47	13,4708	0	Unmethylated	Unmethylated
PANEL_B_01	10,25182	0	Unmethylated	Unmethylated
PANEL_B_02	10,02701	0	Unmethylated	Unmethylated
PANEL_B_03	44,91679	1	Methylated	Methylated
PANEL_B_04	68,0927	1	Methylated	Methylated
PANEL_B_05	6,678102	0	Unmethylated	Unmethylated
PANEL_B_06	13,74234	0	Unmethylated	Unmethylated
PANEL_B_07	15,85766	0	Unmethylated	Unmethylated
PANEL_B_08	14,8438	0	Unmethylated	Unmethylated
PANEL_B_09	76,51168	1	Methylated	Methylated
PANEL_B_10	77,8927	1	Methylated	Methylated
PANEL_B_11	40,00219	1	Methylated	Methylated
PANEL_B_12	12,72482	0	Unmethylated	Unmethylated
PANEL_B_01_1	5,644526	0	Unmethylated	Unmethylated
PANEL_B_01_2	11,49927	0	Unmethylated	Unmethylated
PANEL_B_02_1	11,24161	0	Unmethylated	Unmethylated
PANEL_B_02_2	9,162774	0	Unmethylated	Unmethylated
PANEL_B_03_1	32,23723	1	Methylated	Methylated
PANEL_B_03_2	60,56058	1	Methylated	Methylated
PANEL_B_05_1	6,081022	0	Unmethylated	Unmethylated
PANEL_B_05_2	8,029197	0	Unmethylated	Unmethylated
PANEL_B_06_1	10,82523	0	Unmethylated	Unmethylated
PANEL_B_06_2	16,95693	0	Unmethylated	Unmethylated

Suppl. Table 3. MGMT promoter status assessment. Average indicates mean over 137 selected CpG sites, truth is inferred from EPIC array analysis or pyrosequencing

Sample	Panel	Platform	IDH1	TERTp	MGMT	Methylation_classification	1p loss	19q loss	7 gain	10 loss	EGFR amplification	CDKN2A/B deletion
PANEL_B_09	Rapid_CNS_B	GridION	Yes	Yes	Methylated	O_IDH	Yes	Yes	No	No	No	Yes
PANEL_B_10	Rapid_CNS_B	GridION	No	Yes	Methylated	GBM_RTK_II	No	No	Yes	No	Yes	Yes
PANEL_B_11	Rapid_CNS_B	GridION	No	No	Methylated	GBM_RTK_II	No	No	Yes	Yes	Yes	No
PANEL_B_12	Rapid_CNS_B	GridION	No	Yes	Unmethylated	GBM_RTK_II	No	No	Yes	Yes	Yes	Yes
PANEL_A_47	Rapid_CNS_A	MinION	No	Yes	Unmethylated	GBM_RTK_II	No	No	Yes	Yes	Yes	No
PANEL_B_48	Rapid_CNS_A	MinION	No	Yes	Unmethylated	GBM_MES	No	No	Yes	Yes	Yes	No

Suppl. Table 4. Pathognomonic alterations for new diagnostic samples

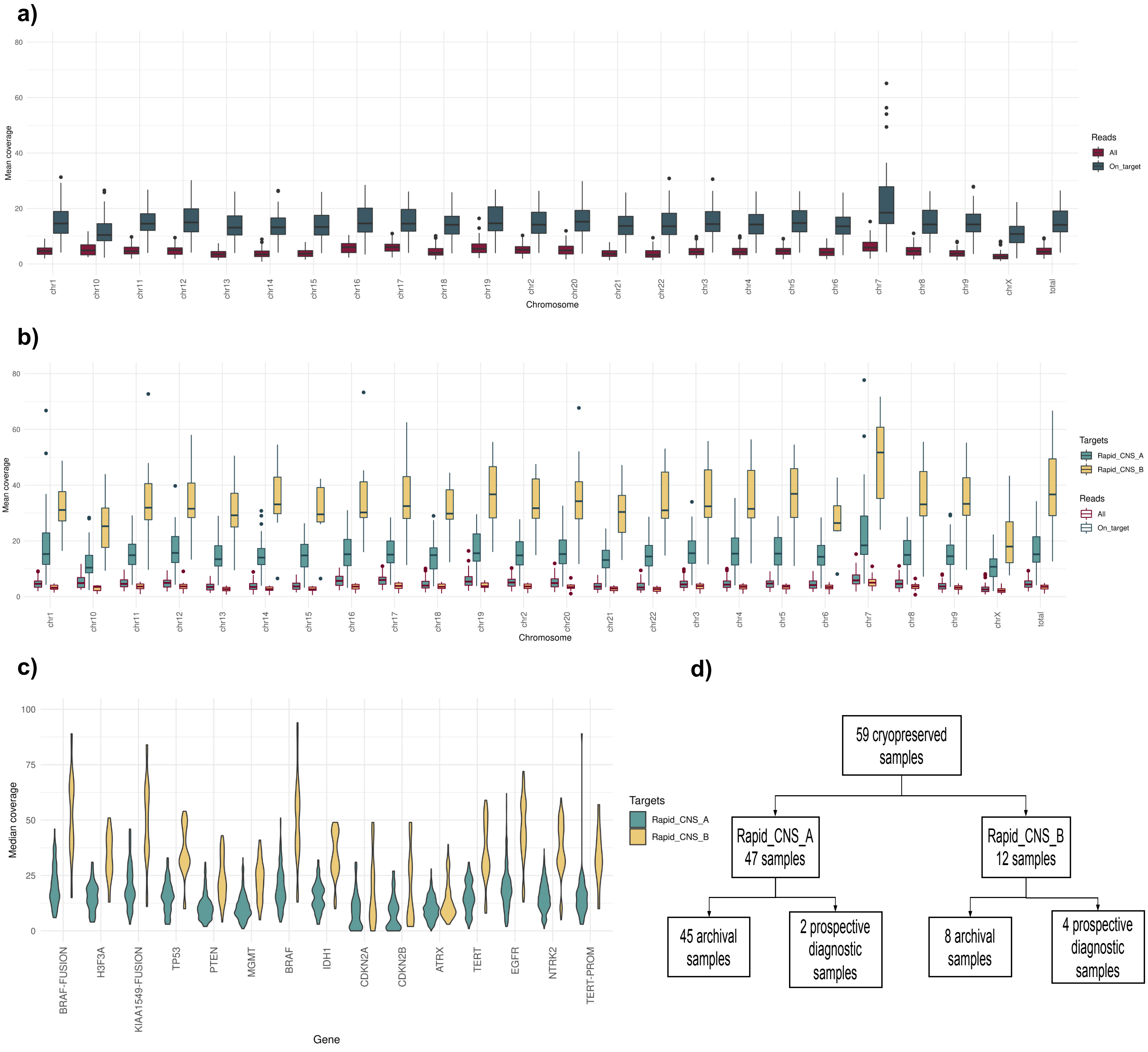

Suppl. Fig 1. a) Mean on-target coverage for 47 samples (PANEL_A_01- PANEL_A_47) sequenced using Rapid_CNS_A. b) Mean on-target coverage for samples sequenced using Rapid_CNS_A vs samples sequenced using Rapid_CNS_B. c) Median coverage over key pathognomonic alterations compared between samples sequenced using Rapid_CNS_A and Rapid_CNS_B. d) Overview of samples.

PANEL_A_02

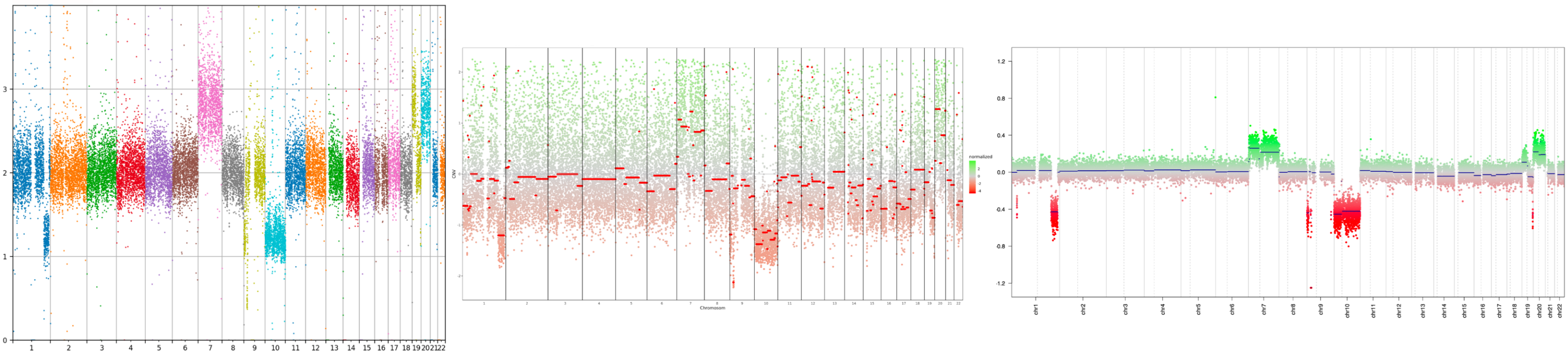

PANEL_A_10

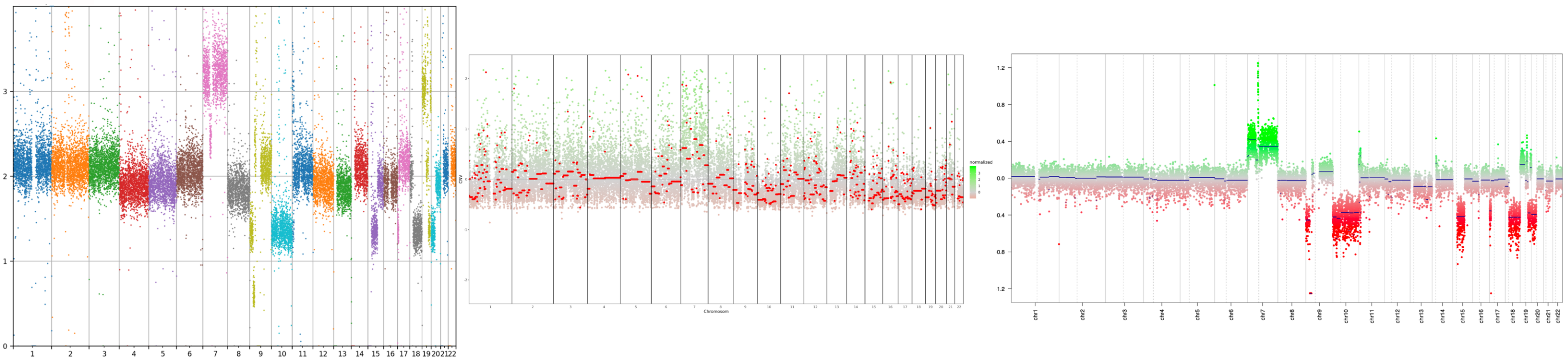

PANEL_A_18

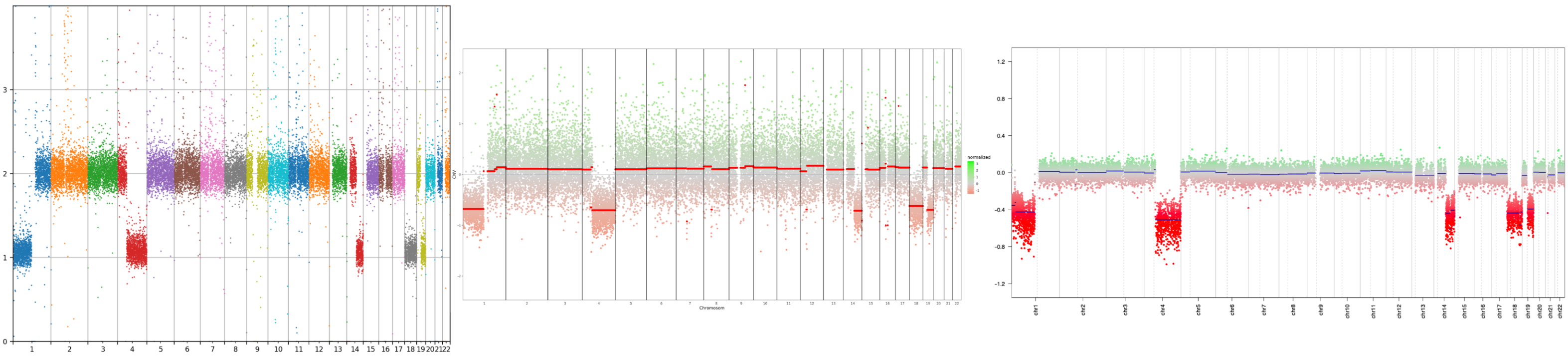

PANEL_A_20

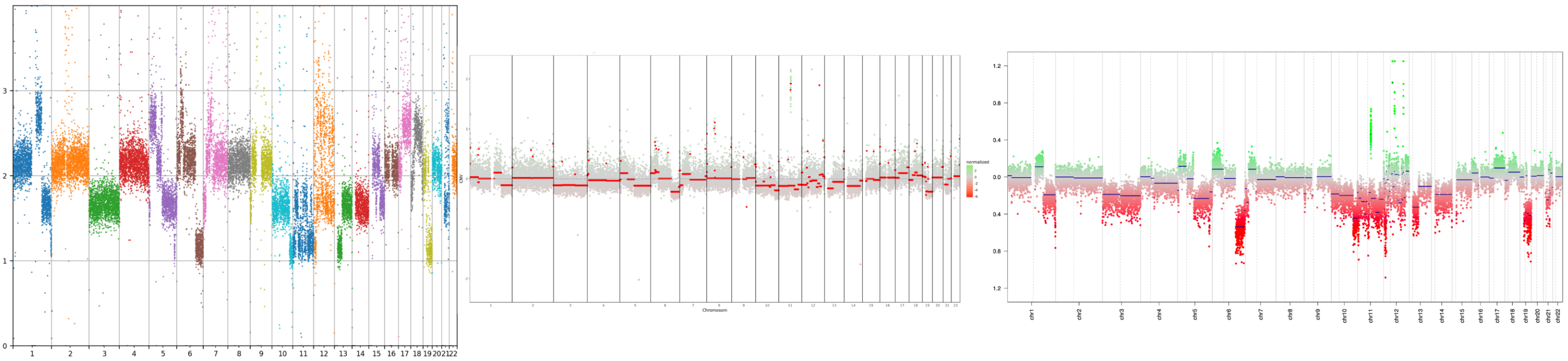

PANEL_B_07

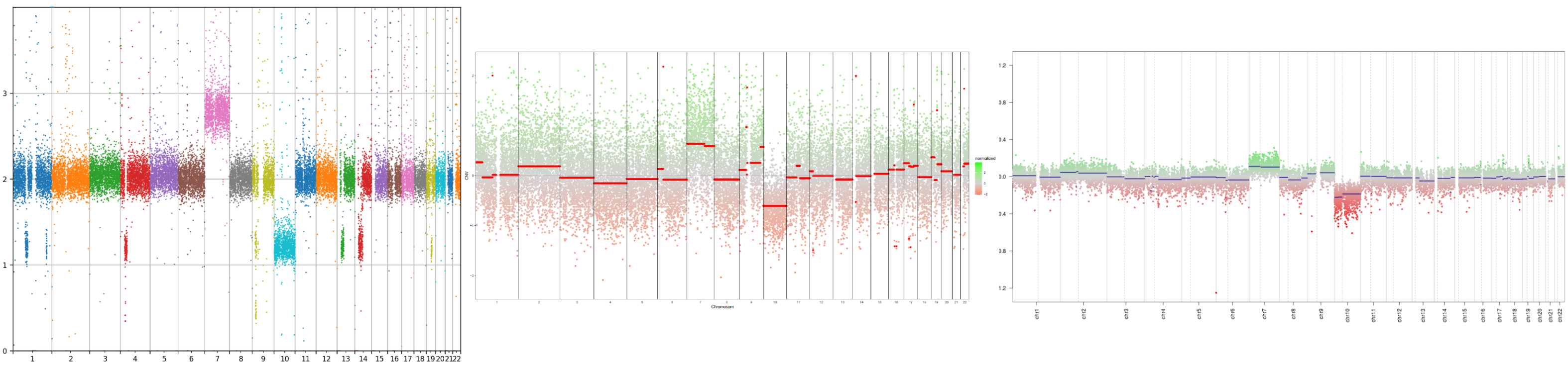

Suppl. Fig. 2 Copy number profiles using Rapid-CNS² (left), NGS panel sequencing (center), EPIC array analysis (right).

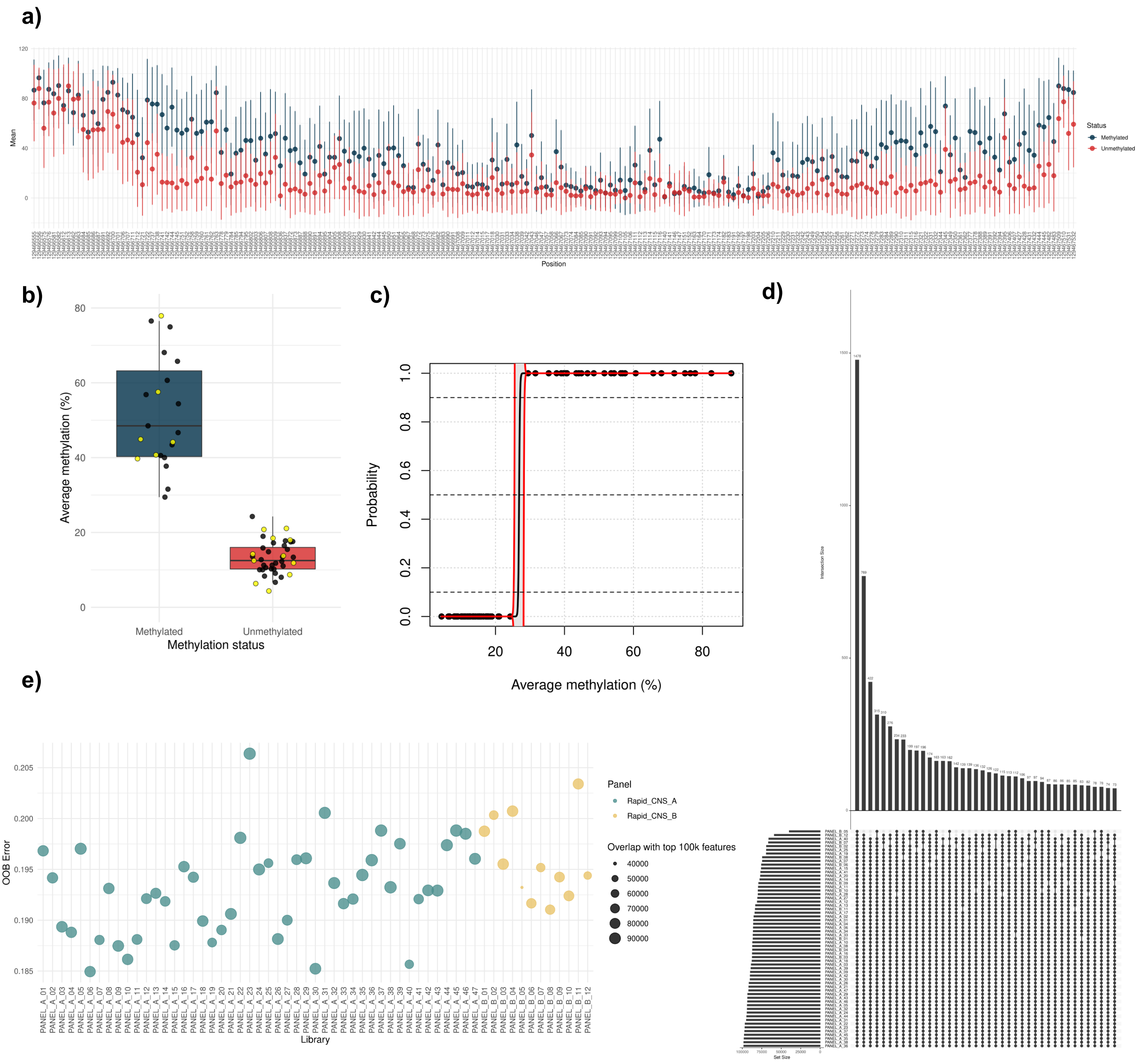

Suppl. Fig. 3 a) Mean methylation over each CpG site in samples with methylated (blue) and unmethylated (red) MGMT promoter regions as reported by EPIC array analysis or pyrosequencing, bars indicate standard deviation. b) Average methylation over 137 filtered CpG sites in methylated and unmethylated cases (training set). Yellow points indicate test samples. c) Probabilities for classification using the proposed logistic regression-based prediction model for MGMT promoter status (0 for unmethylated and 1 for methylated). Gray band indicates the 95% point-wise confidence interval. The averages over 137 CpG sites for methylated and unmethylated samples are shown along y=1 and y=0 respectively. d) Intersection of CpG sites between the 10,000 CpG sites used for training the ad-hoc random forest classifier for each sample. e) Out-of-the-bag error of the ad-hoc random forest classifier trained for each sample. Color indicates whether sample was sequenced using Rapid_CNS_A or Rapid_CNS_B for 72h.

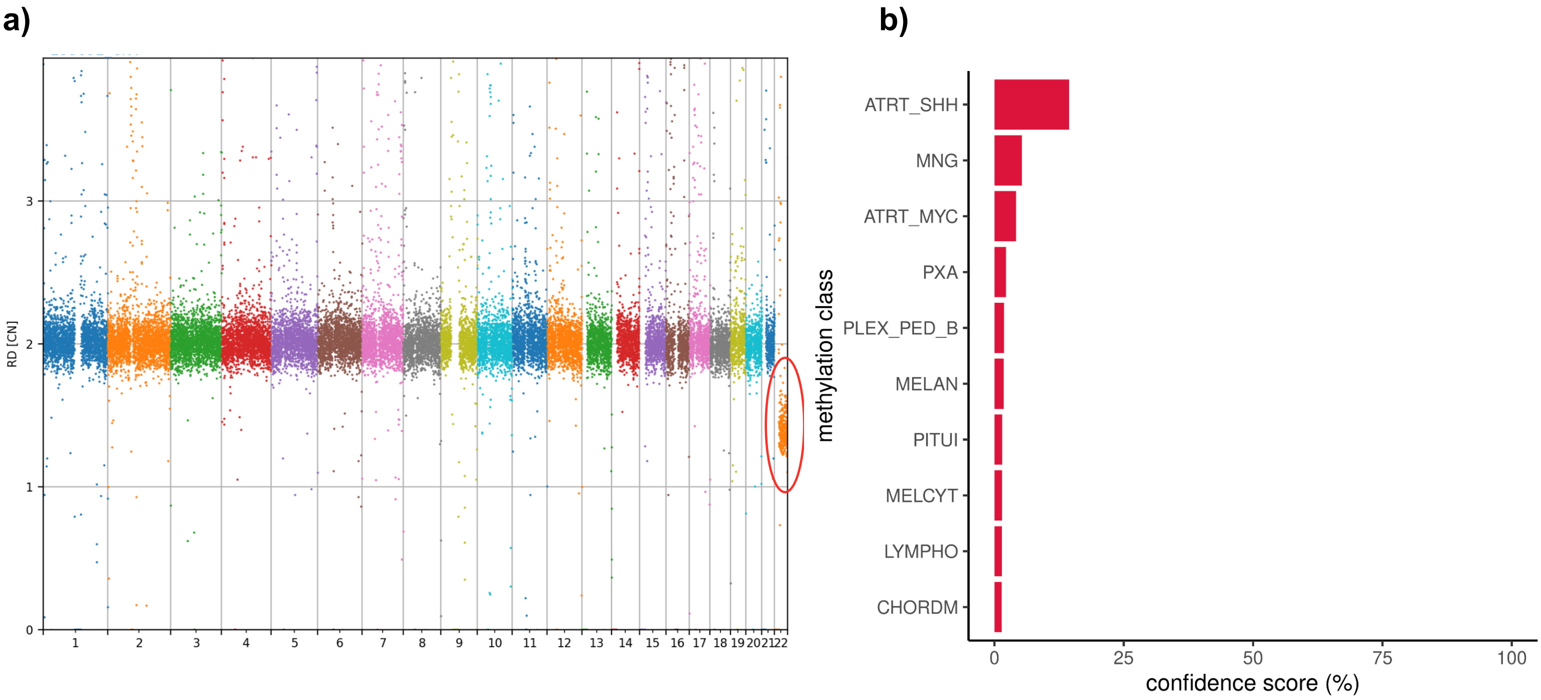

Suppl. Fig. 4 a) Copy number profile (with chr22q deletion highlighted) and b) confidence score for methylation classification for PANEL_B_08.

FOR RESEARCH USE ONLY

HEIDELBERG UNIVERSITY HOSPITAL

Patient name: XXXXXX

Sample ID: PANEL_B_07

Submitted on: XX.XX.XXXX

Reported on: XX.XX.XXXX

Panel: Rapid-CNS^2 (Rapid_CNS_B)

*This report was generated by Rapid-CNS^2 and is intended for research use only. Frozen tumour tissue was prepared and processed using the SQK-LSK110 Ligation Sequencing Kit from Oxford Nanopore Technologies. 160 gene regions from the NPHD panel were targeted using an adaptive sampling approach on the GridION. Data was analysed by the custom Rapid-CNS^2 pipeline. Comprehensive analysis of CNS tumours using Rapid-CNS^2 is a research tool currently under development. It has not been clinically validated in sufficiently large cohorts. Interpretation and implementation of the results in a clinical setting is in the sole responsibility of the treating physician.

Mutations:

Chr	Start	End	Ref	Alt	Func	Gene	ExonicFunc	cytoBand	avsnp147	X1000g2015 aug_eur	cosmic68
chr5	1295228	1295228	G	A	upstream	TERT	.	5p15.33	.	.	.
chr10	89692826	89692826	T	-	exonic	PTEN	frameshift deletion	10q23.31	.	.	ID=COSM1349503
chr10	89692839	89692839	T	G	exonic	PTEN	nonsynonymous SNV	10q23.31	.	.	ID=COSM13135
chr12	57499094	57499094	G	-	exonic	STAT6	frameshift deletion	12q13.3	.	.	ID=COSM1363223
chr12	121434630	121434630	-	TCATT CAT	intronic	HNF1A	.	12q24.31	rs397707647	.	ID=COSM1645167
chr9	87459812	87459812	A	-	intronic	NTRK2	.	9q21.33	rs373383833	0.001	.
chr16	4987050	4987050	G	T	UTR5	PPL	.	16p13.3	rs376539573	0.001	.
chr17	29557466	29557466	A	G	intronic	NF1	.	17q11.2	rs535609733	0.001	.
chr12	69229771	69229771	T	-	intronic	MDM2	.	12q15	rs533467429	0.002	ID=COSM1639229 ,COSM1639228
chr2	46611853	46611853	C	T	UTR3	EPAS1	.	2p21	rs142847751	0.003	.

Pathology evaluation:

Glioblastoma

MGMT promoter:

MGMT promoter methylation status is assessed using a logistic regression based prediction model

Status = **Unmethylated**

Probability = 1.00000

Average = 12.72%

(recommended cut-off is 25%)

Methylation classification:

Methylation-based classification is based on 58,856 CpG sites. The Brier score of this ad-hoc classifier is 19.3%. Using this classifier, the sample has been classified as **glioblastoma, IDH wildtype, subclass RTK II.** This prediction has a confidence score of 76.2%. The calibrated score distribution for the Top 10 entities is given below.

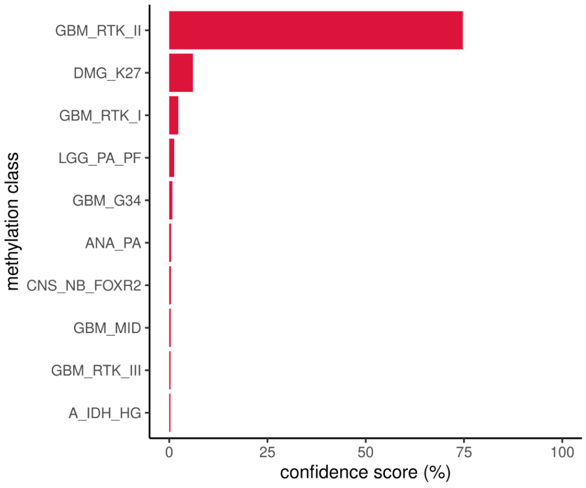

Copy number profile:

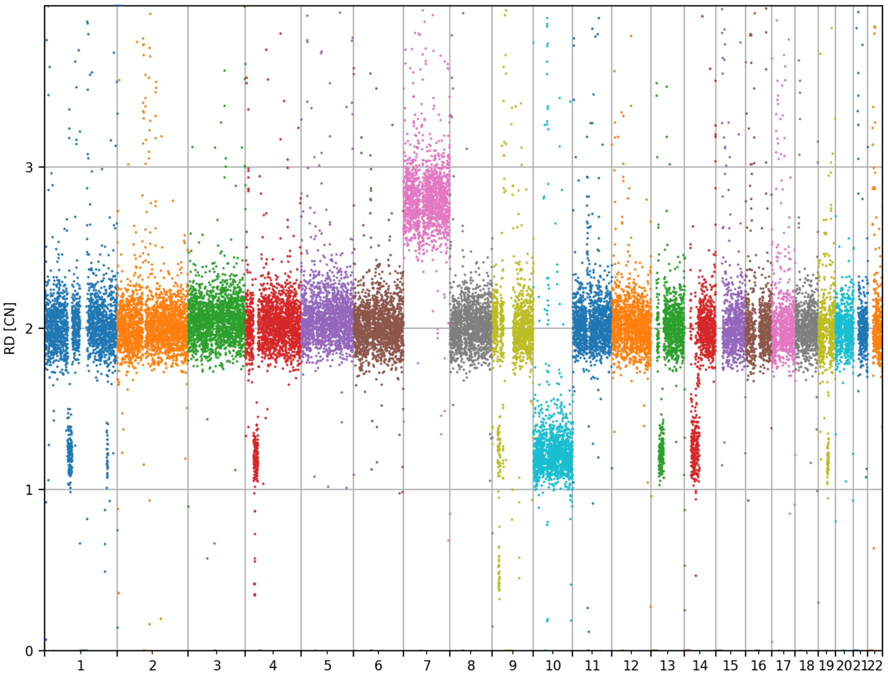

Suppl. Fig. 5 Sample report PDF for prospective diagnostic case PANEL_B_07

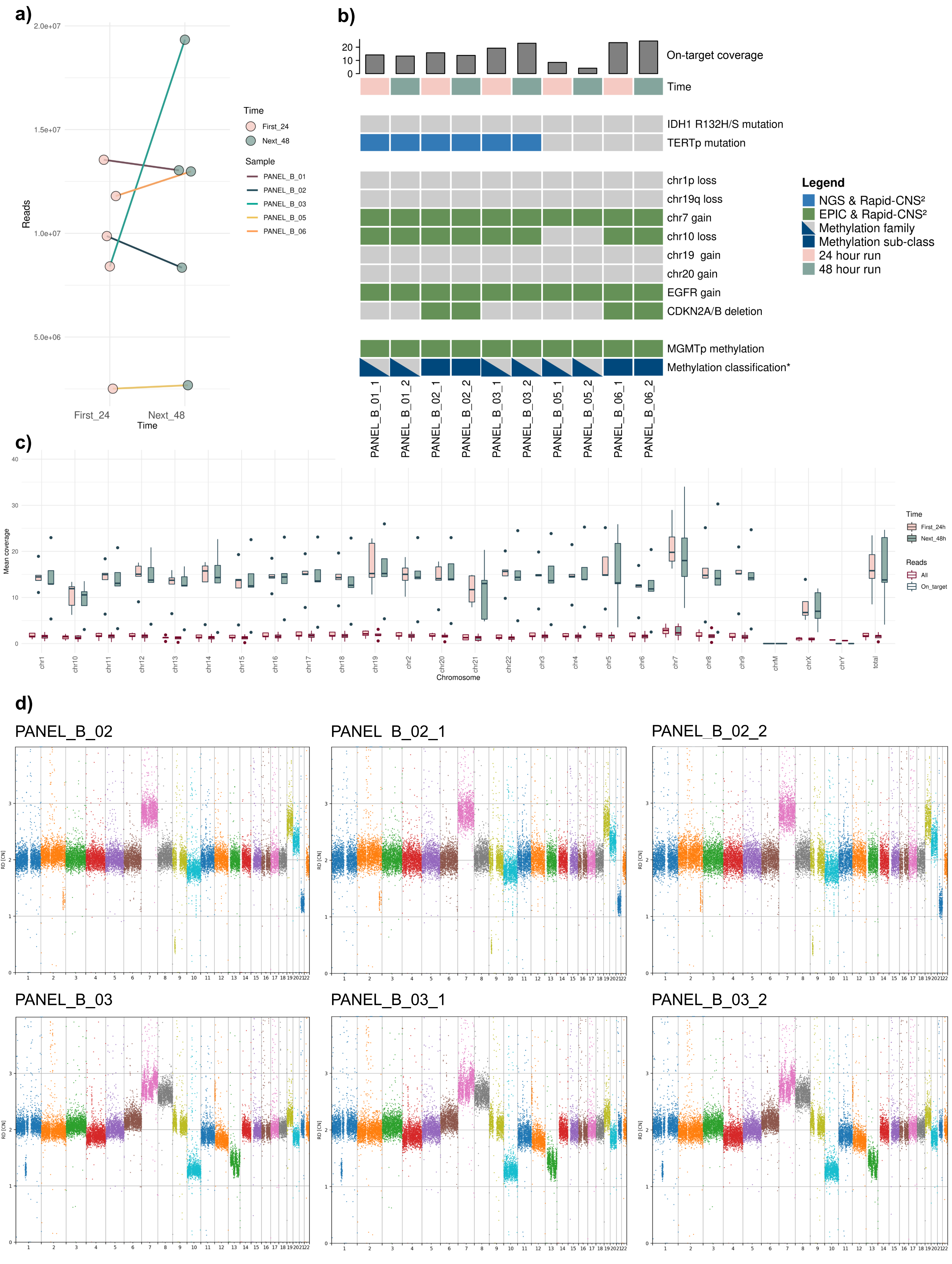

Suppl. Fig. 6 a) Number of reads generated per sample (PANEL_B_01,02,03,05,06) over the first 24h run (left) and next 48h run after flushing and reloading (right). b) Concordance of clinically relevant alterations & classification for samples over the first 24h run (PANEL_X_1) and subsequent 48h run (PANEL_X_2). Annotations same as Fig. 1 d. c) Mean coverage over on target regions vs all regions per chromosome for the first 24h run and next 48h runs. d) Copy number profiles for the 72h run (left), first 24h run (middle) and next 48h run (right).

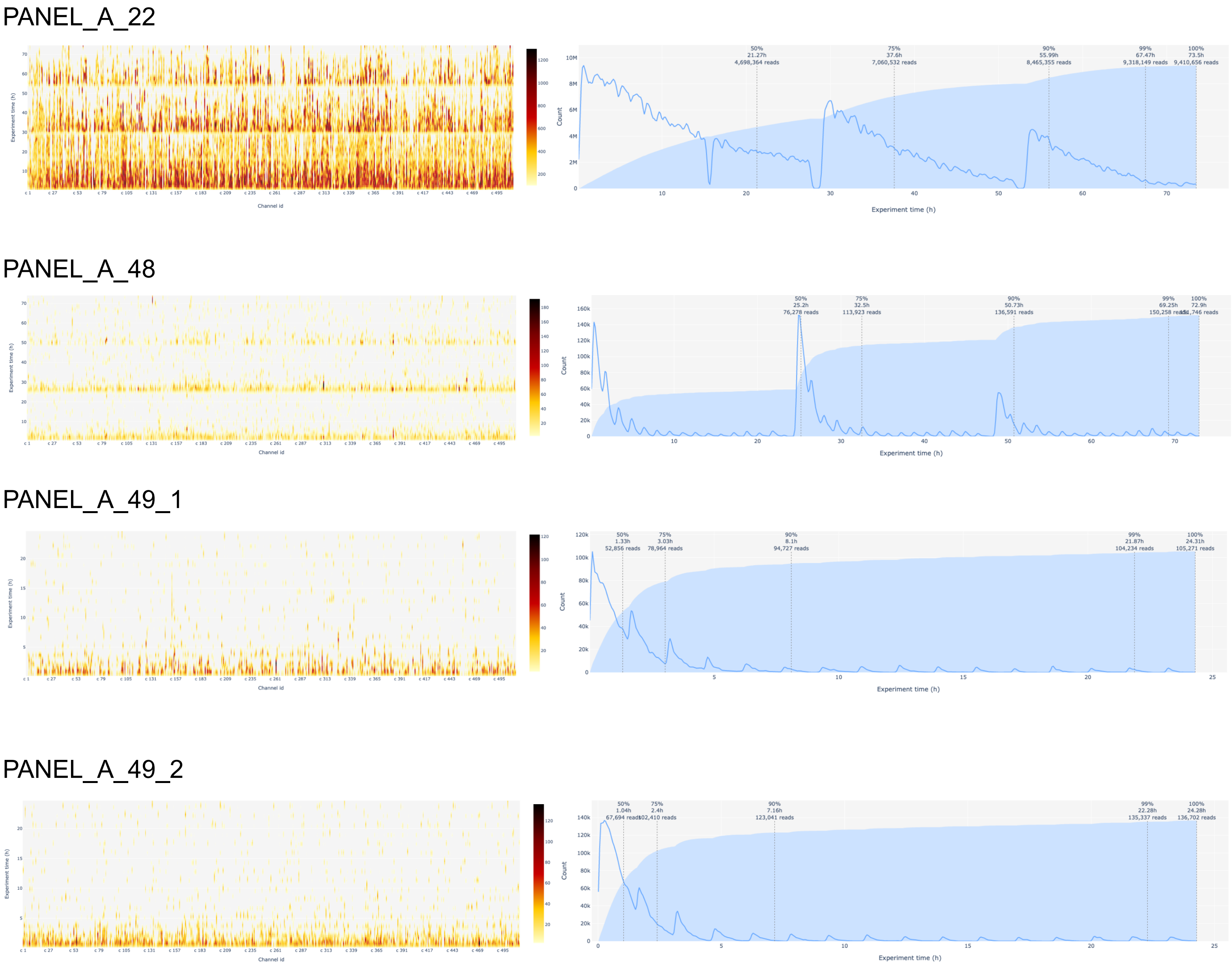

Suppl. Fig. 7 FFPE samples. Channel activity (left) and cumulative read counts over time (right) for a cryopreserved sample (PANEL_A_22) compared to FFPE samples- PANEL_A_48 (72h), PANEL_A_49_1 and PANEL_A_49_2 (24h).
